# Improved cyber-physical system captured post-flowering high night temperature impact on yield and quality of field grown wheat

**DOI:** 10.1038/s41598-020-79179-0

**Published:** 2020-12-17

**Authors:** Nathan T. Hein, Raju Bheemanahalli, Dan Wagner, Amaranatha R. Vennapusa, Carlos Bustamante, Troy Ostmeyer, Meghnath Pokharel, Anuj Chiluwal, Jianming Fu, Dhanush S. Srikanthan, Mitchell L. Neilsen, S. V. Krishna Jagadish

**Affiliations:** 1 Department of Agronomy, Kansas State University, 1712 Claflin Road, Manhattan, KS 66506-5501 USA; 2 Department of Computer Science, Kansas State University, Manhattan, KS 66506 USA; 3 Present Address: Department of Plant and Soil Sciences, University of Kentucky, Lexington, KY 40546 USA

**Keywords:** Plant stress responses, Climate-change impacts

## Abstract

Winter wheat (*Triticum aestivum* L.) is essential to maintain food security for a large proportion of the world’s population. With increased risk from abiotic stresses due to climate variability, it is imperative to understand and minimize the negative impact of these stressors, including high night temperature (HNT). Both globally and at regional scales, a differential rate of increase in day and night temperature is observed, wherein night temperatures are increasing at a higher pace and the trend is projected to continue into the future. Previous studies using controlled environment facilities and small field-based removable chambers have shown that post-anthesis HNT stress can induce a significant reduction in wheat grain yield. A prototype was previously developed by utilizing field-based tents allowing for simultaneous phenotyping of popular winter wheat varieties from US Midwest and advanced breeding lines. Hence, the objectives of the study were to (i) design and build a new field-based infrastructure and test and validate the uniformity of HNT stress application on a scaled-up version of the prototype (ii) improve and develop a more sophisticated cyber-physical system to sense and impose post-anthesis HNT stress uniformly through physiological maturity within the scaled-up tents; and (iii) determine the impact of HNT stress during grain filling on the agronomic and grain quality parameters including starch and protein concentration. The system imposed a consistent post-anthesis HNT stress of + 3.8 °C until maturity and maintained uniform distribution of stress which was confirmed by (i) 0.23 °C temperature differential between an array of sensors within the tents and (ii) statistically similar performance of a common check replicated multiple times in each tent. On average, a reduction in grain-filling duration by 3.33 days, kernel weight by 1.25% per °C, grain number by 2.36% per °C and yield by 3.58% per °C increase in night temperature was documented. HNT stress induced a significant reduction in starch concentration indicating disturbed carbon balance. The pilot field-based facility integrated with a robust cyber-physical system provides a timely breakthrough for evaluating HNT stress impact on large diversity panels to enhance HNT stress tolerance across field crops. The flexibility of the cyber-physical system and movement capabilities of the field-based infrastructure allows this methodology to be adaptable to different crops.

## Introduction

Winter wheat (*Triticum aestivum* L.) is an important staple cereal and a major source of calories for a large proportion of the world’s population. Wheat production has increased over the last two decades and currently competes with rice (*Oryza sativa*) to be the second most produced cereal in the world, after maize (*Zea mays*)^1^. Despite the progress made, by 2028 worldwide production of wheat is expected to increase by 1.01%, while the demand is projected to increase at 1.13% due to a substantial increase in world population^2,3^. Along with the inherent need to increase production for a growing population, the changing climate also threatens the future yield potential of food crops including wheat. The Intergovernmental Panel on Climate Change^4^ has concluded that the mean surface temperature of the Earth will continue to rise this century and that heat waves will continue to occur more frequently, with more intensity and with each event lasting for longer duration. This increase in global mean surface temperature is being driven by an increase in the average daily minimum temperature, which is rising at a quicker rate than the average daily maximum temperature^5–8^. Studies related to high night temperature (HNT) stress on different crops have increased recently (between 2010 and 2020) with many of these focused on rice^9–15^. A comprehensive study conducted at the International Rice Research Institute in the Philippines showed that the mean maximum temperature between 1979 and 2003 rose by 0.35 °C while the annual mean minimum temperature rose by 1.13 °C. This study revealed that grain yield in rice was reduced by 10% for every 1 °C increase in average seasonal minimum temperature^16^. Other studies have also shown detrimental effects of HNT stress in different crops including sorghum^17^, cotton^18–20^, soybean^21^, corn^22^ and wheat^23,24^.Using six different night temperatures (between 15 and 27 °C) from heading until maturity in controlled environment growth chambers, a threshold of 23 °C was identified to significantly reduce grain filling-duration and yield in winter wheat genotypes^24^. Studies on HNT stress in winter wheat have been mainly accomplished by using controlled environment growth chambers^23–26^ or small chambers under field conditions^27–29^. These facilities would have inherent difficulty in capturing large genetic diversity to HNT stress and chambers in particular would be challenged by differences in light, wind speed and humidity compared to field conditions, resulting in altered microclimate. Until 2019, there did not exist a large mobile field-based infrastructure with the ability to impose controlled HNT stress on crops. In response to this need, a field-based prototype was constructed which facilitated successful imposition of HNT stress throughout the grain-filling period in winter wheat^30^. A stable HNT stress of + 3.2 °C throughout the grain-filling period resulted in a 5% reduction in yield (averaged across 12 cultivars) per °C increase in night temperature, supporting findings from Garcia et al.^28,30^. Finding solutions by utilizing diversity panels or mapping populations to address the negative impact and minimize the damage caused by HNT requires effective upscaling of the prototype presented in Hein et al.^30^. Necessary components which required upscaling to provide the ability to impose stress on large diversity panels or mapping populations include the heat tent structure and heating system along with a brand new cyber-physical system (Table 1). In addition, grain size, in particular grain width, was demonstrated to be a key trait that translated to lower yield and poor grain quality i.e. increased protein and lipids at the cost of starch under HNT stress^24,31^. This information on grain protein and starch imbalance has been captured under controlled environment growth chambers, but whether the same results can be extended to field grown wheat is not known^24^. Hence, the objectives of the study were to (i) design and build a new field-based infrastructure and test and validate the uniformity of HNT stress application on a scaled-up version of the prototype presented in Hein et al.^30^; (ii) improve and develop a more sophisticated cyber-physical system to sense and impose post-anthesis HNT stress uniformly until physiological maturity, within the scaled-up tents; and (iii) determine the impact of HNT stress during grain filling on the agronomic and grain quality parameters including starch and protein concentration.

**Table 1:** Improvements in large scale field-based heat tents and cyber-physical system compared to the prototype presented in Hein et al.^30^, for phenotyping impact of high night-time temperature stress. N/A—Not applicable

System component	Feature	Hein et al. ^30^ prototype heat tent	Large scale mobile heat tent
Heat tent structure	Dimensions	7.2 m × 5.4 m × 3.0 m	9.1 m × 14.6 m × 4.4 m
	Number of genotypes	12	320
	Planting height	Could only accommodate wheat or small row crops	Can accommodate small rows crops, sorghum, maize, pearl millet etc
	Ventilation	Small roof vent and manual sidewall roll-ups	Roof, sidewalls, and end-walls mechanical roll-ups
	Mobility	Hand carried by 12 people	Built on skids—moved through towing with a tractor
	Number of heat tents	3 heat tents with control plots under ambient open field conditions	3 heat and 3 control tents
Heating system	Heater	Small electrical heater	Energy efficient propane heater
		Tank top propane heater	N/A
	Heat distribution	Built in fan on heater	Additional blower fan on heater with convection tubing allowed efficient and uniform heat distribution
	Ventilation	N/A	Direct ventilation of combustion exhaust to the exterior of the tent
	Fans	Box fan above tank top propane heater	Two powerful circulation fans
Cyber-physical system	Basic function	Line voltage disruption	Operated multiple relays to act as thermostat
	Sensor system	Single sensor indoors and outdoors	Six sensor temperature arrays
	Communication	N/A	Wireless communication between control and stress at 1 min interval
	Additional sensor capabilities	N/A	CO_2,_ relative humidity, and rain sensors
	Heat distribution analysis	N/A	Capable of mapping heat distribution and uniformity across the entire tent
	Control environment	Ambient conditions not accounting for tent structure	Ambient conditions but within a tent to isolate unaccounted external variables

## Materials and methods

### Field infrastructure

**Figure 1:**
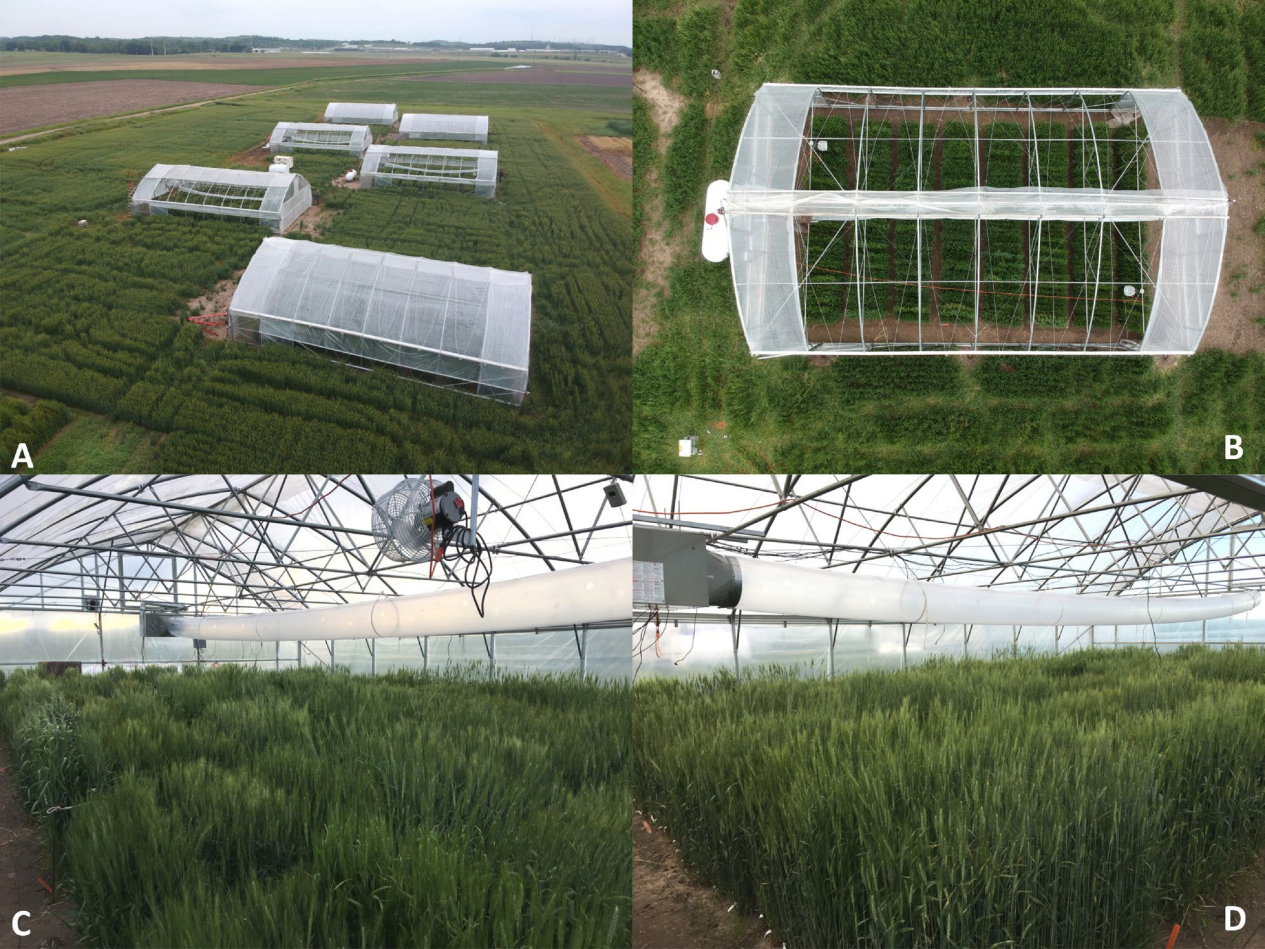
An overview of field and tent layout. (**A**) An over-head view of all six tents with the three control tents (two in the farthest background and one in the closest foreground) in their night setting with the roof closed and the sidewall lowered and three stress tents (centrally located) in their day setting. (**B**) Overhead view of a stress tent with a propane tank on the far left and the roof opened to its daytime setting. Eight blocks of 40 individual rows shown along with circulation fans in the upper-left and bottom-right portion of the interior of the tent. (**C**) Interior view of a stress tent looking towards the heater with the circulation fan in the foreground and propane heater in the background. The convection tubing extended from the propane heater throughout the entire tent to distribute the heated air uniformly. (**D**) Interior view of a stress tent looking from the heater towards the opposite side of the tent. The convection tubing is seen all the way extended to the endwall and the roof and sidewalls lowered in their night setting. An additional figure indicating each component within and outside the tents is presented in Supplementary Fig. 1.

The custom designed and movable heat tents are 9.1 m wide, 14.6 m long and 4.4 m tall (Four Season Tools, Kansas City, MO, USA; Table 1, Fig. 1; Supplementary Fig. 1). The structures had 1.9 m tall sidewalls that were joined with a vertical truss every 1.8 m along the length of the building. The combination of taller sidewall piping with the roof trusses allowed for a 2.4 m working height below the bottom chord of the roof trusses. The buildings were designed to study the impact of HNT stress on a wide variety of row crops including corn and sorghum which was not possible in the previous iteration of the system (Table 1). Each heat tent was built on top of 15.2 m long skids with integrated ski tips on the ends allowing the tent to be moved in either direction utilizing a tractor. The end walls were built with retractable studs to be removed during movement which gives the heat tent the ability to be placed over the crop during the target developmental stage/s, otherwise allowing the crop to grow in normal field conditions. Due to its movement capabilities, the structure was braced throughout to prevent bending or misshaping.

The tents were enclosed with a 6 mm polyethylene plastic with a 92% light transmission according to the manufacturer (Berry Global Plastics, Evansville, IN, USA). A motorized roll-up system (Advancing Alternatives, Lancaster, PA, USA) on all the sidewalls, endwalls and roofs were installed to allow the heat tents to be as open as possible throughout the day as to not impose a high day temperature stress, similar to the principle published in rice^10,12^. This system utilized 24 VDC motors with guide bars that, when initiated, automatically rolled the plastic up to the day setting and allowed for a more open and truly ambient daytime condition when compared to Hein et al.^30^ (Table 1, Fig. 1).

To increase the temperature within the tents designated for elevated stress, a heating system was designed to operate automatically overnight. A Modine HDB100 Hot Dawg propane heater (Ferguson Plumbing and Heating, Manhattan, KS, USA) was installed in each stress tent utilizing square steel and steel rods between the end wall and first vertical truss (Fig. 1). This unit has an 82% efficiency rating and outputs 82,000 BTU/hour at 781 FPM. The heater was augmented with a duct transition to allow the attachment of convection tubing. The tubing itself was 45.7 cm in diameter and 13.7 m in length (Fig. 1; Supplementary Fig. 1). The convection tubing was punctured every 1.2 m with round openings with a diameter of 5.7 cm at 3 o’clock and 9 o’clock to force the heated air to escape parallel with the field. The heaters were supplied with propane via individual 1829.7 L tanks (Propane Central, Clay Center, KS, USA). Two 30.5 cm horizontal air flow fans (J&D Manufacturing, Eau Claire, WI, USA) with an air flow rate of 1,020 CFM were hung from the bottom chord of the trusses in opposite corners to evenly circulate the heated air (Fig. 1; Supplementary Fig. 1). The larger heating system with convection tubing and dual circulation fans allowed for a single heater to completely and equally impose stress while, along with the addition of the combustion exhaust, created a safer and more controlled environment than previously capable in Hein et al.^30^ (Table 1).

The control tents were outfitted with a very similar set up without the implementation of heat. To imitate the same sensation of air movement on the plants as in the stress tents, a 45.7 cm power tube fan (Coolair, Jacksonville, FL, USA) was installed and convection tubing ran with the same hole set up as the stress tents. The same horizontal air flow fans were also installed to circulate the air throughout each of the three control tents.

To operate the tents, a Caterpillar XQ30 electric generator (Foley Power Solutions, Topeka, KS, USA) was placed centrally located in the field. The generator output 30 KW/38 KVA. A 3785.4 L diesel tank (Capital City Oil, Topeka, KS, USA) was positioned very near to the generator and was outfitted with a battery operated 12-V DC pump with an output of 75.7 L per minute, hose and nozzle for refueling the generator, similar to Hein et al.^30^. Two 50 amp spider boxes were wired to the generator with 30.5 m twist-lock cords to allow distribution of electricity to all tents. Electricity was then distributed to each tent using various lengths and gauges of extension cords depending on the size of load and length of run.

### Temperature controller system

#### Overall description/functionality

The thermostat controller system was newly designed to monitor the temperature within each tent, average the temperature sensor array readings and wirelessly transmit the temperature from the control tents to their corresponding stress tents for comparing the control and stress environments. When the temperature within a stress tent was less than 4 °C warmer than the corresponding control tent, the system engaged the propane heater to increase the interior temperature of the stress tent. If the stress tent was 4 °C or more above the control tent, the heater was not engaged and the temperatures continued to be monitored. This improved functionality as well as having the capability to be equipped with multiple different sensor types to gather data on different environmental variables simultaneously resulted in a much more robust and stable system (Table 1).

**Figure 2:**
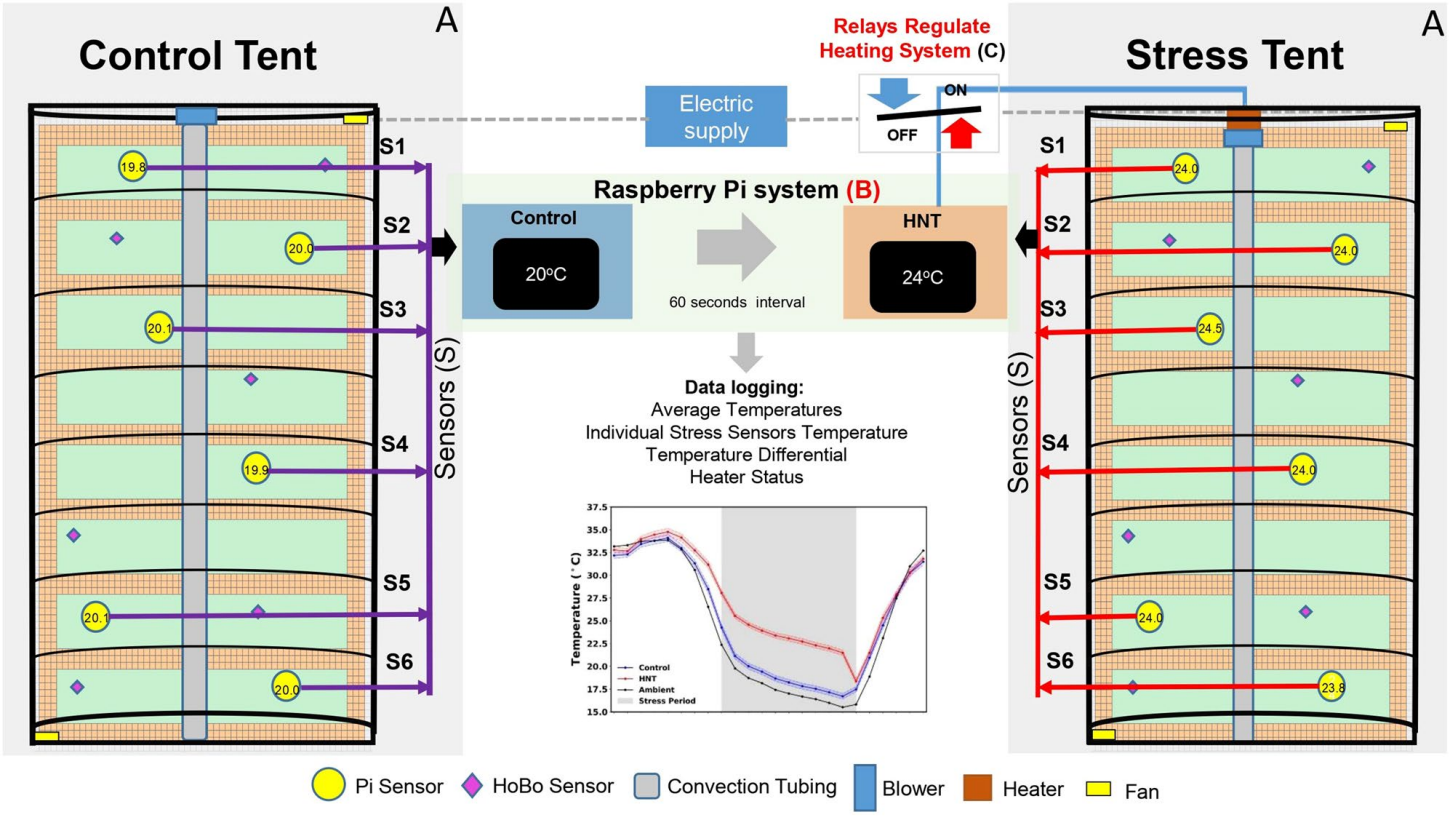
Diagrammatic presentation of a paired control tent with a stress tent, depicting the operation. (**A**) A detailed interior view of the stress and control tent; see Supplementary Fig. 1. (**B**) A detailed explanation of Raspberry Pi System and its wiring is presented in Supplementary Figs. 2–4, Supplementary File System Details and Code and Supplementary Table 2. (**C**) An overview of the relay system and their wiring can be found in Supplementary Figs. 3 and 4.

The thermostat controller system consisted of a Raspberry Pi (Raspberry Pi Foundation, Cambridge, UK), six MCP9808 temperature sensors (Adafruit, New York City, NY, USA) and one DS3231 Real-Time Clock (RTC) module (Adafruit) for each tent. In addition, stress tents contained a four-channel Solid State Relay (Keyes KY-019 Relay Module, Songle Relay, Yuyao City, Zhejiang, China) for controlling the heater (Supplementary Figs. 2–4) and one MH-Z19 carbon dioxide sensor (Zhengzhou Winsen Electronics Technology Co., Ltd., China) for monitoring carbon dioxide levels. An electrical junction box was used to water- and dust-proof the Raspberry Pi, RTC and the Solid State Relay (Supplementary Fig. 2). Each individual temperature sensor and the CO_2_ level within the tent was read every minute and logged into a CSV file with accurate timestamps from the RTC module. The overall system health was logged to file for troubleshooting and verification purposes. A systematic view of the overall spread of the temperature sensors within the tents, the integration of wireless flow of information on temperature between control and heat tents is illustrated in Fig. 2.

#### Design philosophy

Autonomy, versatility, ease of use and robustness were factored into the system design. When the system was powered on, the controller automatically initialized itself and began reading temperature sensors and logging data to the file automatically. The code base allows users to define their own implementations for interacting with different types of sensors i.e., different heaters can be used with few changes to the code (Supplementary File System Details and Code). Users can interact with the system using simple Linux commands to verify that everything is operating correctly and to retrieve the log files. The system does not crash when a sensor fails to read, it instead attempts to reboot after a set number of consecutive read errors and, after a maximum number of reboots, remains online to continue the experiment.

Software failures were typically resolved by rebooting the Raspberry Pi. If the temperature sensors were detected on the I2C bus but were not read, the baud rate was reduced further, this allowed more time for the signals to propagate down the leads to be read by the Pi. If none of the sensors were being read, then each individual sensor connected to the system was manually inspected for short circuited pins, any found were removed from the system and replaced with new components and tested after installation. For more detailed description and explanation of the temperature control system hardware, components and codebase, as well as a detailed list of components and their sources for the entire field-based infrastructure system see Supplementary File System Details and Code, Supplementary Figs. 2–4, and Supplementary Tables 1 and 2 for Tent and Raspberry Pi Components.

### Operation of the tents and stress imposition

The HNT stress was imposed at night from 8:00 PM until 6:00 AM beginning after all genotypes reached 50% anthesis and continued until physiological maturity. The experimental process began at 6:30 PM with rolling down the roofs of the stress tents and then the side and end walls. After the stress tents were closed, the control tents roofs were closed and the side and end walls were lowered to 20 cm above the baseboard to allow ambient air to circulate through the tent (Fig. 1; Supplementary Fig. 1). By 7:00 PM the generator was activated and all control tents were provided power immediately to initialize their systems. After the cyber-physical systems were running in the control tents, the stress tents were turned on consecutively to allow for monitoring the initiation of the propane heaters. The stress tents were deemed operational by physically viewing the operation of the propane heaters and the entire cyber-physical system was judged operational through wireless analysis of the Raspberry Pi. The system then achieved the indicated differential temperature in the heat tents by 8:00 PM to start the overnight stress period.

The system began to be shut down at 6:00 AM by first wirelessly collecting the data from the Raspberry Pis and then consecutively removing the power from the Raspberry Pis within the stress tents. Without electricity, the call for heat from the cyber-physical system would cease and the heaters would go through their shutdown procedure. Once all heaters had processed through their shutdown procedure, the generator was turned off which removed power from the entire system. The propane was then turned off both within the heater and on the tank for safety redundancy. The roofs, endwalls and sidewalls were raised to their daytime ambient setting on all tents concurrently with the system shutdown procedure.

Each tent was monitored with multiple sets of sensors to capture temperature, relative humidity and carbon dioxide. The Raspberry Pi system itself utilized six temperature sensors, spread across the tent, with a sensitivity of ± 0.25 °C which monitored the tents throughout the night while the system was running and recorded data every minute (Fig. 2). This new and expanded sensor array not only gathered a more accurate representation of current temperature levels within the tents, but also allowed for post-analysis of the uniformity of heat distribution through individual sensor recordings (Table 1). The Raspberry Pi system in the stress tents also recorded carbon dioxide levels with one MH-Z19 carbon dioxide sensor, with a sensitivity of 50 ppm + 5% reading value, randomly placed within the tent 25 cm above canopy level. Each tent was also equipped with two HOBO UX 100–011 temperature/relative humidity data loggers (Onset Computer Corp., Bourne, MA, USA) to record relative humidity with an accuracy of ± 2.5%, recorded once every 15 min. Finally, the heat tents were outfitted with two HOBO UA 002-64 Pendent data loggers (Onset Computer Corp., Bourne, MA, USA) which recorded temperature at a sensitivity of ± 0.53 °C and light intensity. These data loggers recorded data at 15 min intervals throughout the course of the experiment (Fig. 2).

### Crop cultivation

The tents and connected cyber-physical systems were tested in a field-based experiment at the Kansas State University Agronomy North Farm in Manhattan, Kansas (39° 12′ 47.3″ N 96° 35′ 35.0″ W). This experiment utilized the same set of genotypes tested in the prototype^30^, which were included as a subset within the larger diversity panel (n = 320; aimed at Genome Wide Association Studies [GWAS]). The selection included five wheat varieties extensively grown in Kansas (Everest, Larry, SY-Monument, WB-4458 and WB-Cedar), five common breeding lines from the Kansas wheat breeding program (Jagger X060724, KS 070736K-1, KS 070729K-26, KS 070717M-1 and P1 X060725) and two exotic varieties (Tascosa and Tx86A5606) which have been previously shown to have differential responses to heat stress^23,30,32^. As a part of the larger study, each heat and control tent had eight blocks accommodating all 320 accessions for GWAS analysis, with 40 rows each per block. Each genotype was planted in a single row in each of the three control and heat stress replications with the exception of Everest. This widely used wheat variety in Kansas was used as a check-line to evaluate equal distribution of the heat stress within and between the tents. Each of the eight blocks in all six tents contained a row of Everest, resulting in eight rows of Everest per tent, distributed randomly across blocks.

The space within the tents was measured and marked at 8.2 × 10.7 m. Each tent contained eight blocks containing 40 rows with each row measuring 1.2 m and spaced 19.1 cm apart and with 0.5 m alley between blocks. The rows were trenched to a depth of 3.8 cm using a tool with eight equally spaced prongs to ensure exact and equal depth and spacing throughout the blocks and tents. The trenches were hand planted at 88 seeds per row or 383 seeds/m^2^ or at a rate of 4.45 million seeds per hectare. The seeds were treated before planting with a mixture of 50% Cruiser Maxx Vibrance Cereals (Sedaxane, Difenoconazole, Mefenoxam, Thiamethoxam), 43.75% water and 6.25% Cruiser 5FS (Thiamethoxam) at a rate of 0.33 mL/50 g of seeds and the plots were hand planted on October 23, 2018. Prior to planting 47.07 kg N ha^−1^ (urea ammonium nitrate solution) was applied to the field. On 25th March 2019, 0.88 L ha^−1^ of MCPA herbicide (2-methyl-4-chlorophenoxyacetic acid) and 2.19 cL ha^−1^ of Finesse Cereal and Fallow herbicide (Chlorsulfuron, Metsulfuron Methyl) were applied followed by 57.16 kg N ha^−1^ (urea ammonium nitrate solution) on 26th March, 2019. On 14th May 2019, 0.49 L ha^−1^ of Approach Prima fungicide (Picoxystrobin Methyl, Cyproconazole) was applied to the plots to manage Fusarium Head Blight and the custom built tents were pulled over the plots on 15th May, 2019.

The stress period for the experiment began on May 26th, 2019 as all 12 genotypes had reached 50% anthesis by May 24th, 2019 and continued throughout the grain-filling period until physiological maturity. Hence, the flowering phenology between the 12 genotypes was very narrow i.e., just 2 days and so had no confounding effect on the results. The plots were irrigated with 1325 L of water per tent or 15.1 L m^−2^ on 12th June, 2019 and on 18th June, 2019 to avoid water-limited stress. The plots were irrigated minimally due to an unusually wet spring at the experimental site. The total precipitation for the month of May at the experimental site was 29.1 cm, which is 17.8 cm above the normal precipitation amount. June had a nearly average precipitation totaling to 11.83 cm which was only 1.09 cm below the normal (National Weather Service, https://w2.weather.gov/climate/index.php?wfo=top).

### Agronomic observations

#### Yield and yield components

For grain yield and yield components, a 0.5 m central strip of each genotype, including the eight rows of Everest check-lines in each tent, was hand harvested at physiological maturity (Feekes 11.4). Maturity was determined by daily evaluation of a sample seed set and whether a thumbnail could dent the seed. Spikes were immediately removed from the biomass and dried at 40 °C for 96 h. The biomass was dried separately at 60 °C for 168 h. Spike weight was taken and then the spikes were threshed using an LD 180 Laboratory Thresher (Wintersteiger, Ried im Innkreis, Austria) and grain yield and yield components were recorded. To ascertain any possible impact of Fusarium Head Blight, yield and yield components were also calculated by utilizing the average seed weight of non-infected seeds for each sample. After threshing, the seeds from each sample were manually separated into infected and non-infected categories and the non-infected seeds were then counted and weighed. The non-infected seed sample count and weight was then used to calculate average single seed weight for the non-infected seeds. This average single seed weight was then used to obtain total sample seed weight by multiplying average single seed weight of non-infected seed by the total number of seeds in the harvested sample (including both infected and non-infected seeds). The extrapolated data was highly correlated with the non-categorized whole-sample results with the differential (HNT as a percentage of control) R-Squared values of 0.92, 0.96 and 0.93 for 200 kernel weight, grain yield per m^2^ and harvest index, respectively (Supplementary Fig. 5G,H,I, respectively). Based on the above finding, the non-categorized whole-sample data for yield and yield components were used for analysis of grain yield per m^2^, 200 kernel weight and harvest index (grain yield/total above ground plant weight including grain yield). The dried biomass weight was recorded for calculating harvest index.

### Grain protein and starch concentration

For quantification of grain protein and starch, samples from all twelve genotypes were evaluated utilizing wet chemistry. Grain samples were ground in an 8000 M SPEX Mixer/Mill grinder (SPEX Industries Inc., Metuchen, NJ, USA). A portion of the ground sample was analyzed at the Kansas State Soil Testing Lab (Manhattan, KS, USA), which used a LECO TruSpec CN combustion analyzer to obtain nitrogen concentration on a percent weight basis. This nitrogen concentration was then multiplied by 5.7 to calculate the grain protein concentration^33^. The remaining portion of ground sample was tested for starch concentration utilizing a total starch hexokinase kit (K-THSK, Megazyme, Wicklow, Ireland) as detailed in Impa et al.^23^.

### HNT effect on emergence and seedling vigor

To test HNT effect on the next generation emergence and seedling vigor, harvested seeds were used to quantify these parameters in controlled environment growth chambers. Each plastic tray had six rows with each row having eight individual cells. Each individual cell measured 5.1 cm in diameter and depth. Seeds from all 12 genotypes and both HNT and control were planted randomly in rows, with a single seed in each cell. Each genotype was replicated thrice (i.e. 3 rows = 24 seeds per genotype and treatment). The cells were filled with three parts Vermiculite #3 (Hummert International, Topeka, Kansas, USA) and one part Perlite Hort Grade Coarse (Hummert International, Topeka, Kansas, USA). The soil was dampened with water prior to planting and the seeds were planted at 1.3 cm depth. The trays placed on flat holder trays were moved to a controlled environment growth chamber set at 28/15 °C (actual 28.4/15.3 °C; day/night) with a four hour transition period between day and night and with a photoperiod of 16/8 (light/dark). The flat trays allowed for 1 cm depth of standing water at the bottom ensuring ample water availability throughout^24^. Seedling emergence counts were recorded daily at 4:00 PM and used to calculate total emergence percentage and emergence index^34^. Seedlings were uprooted 14 days after planting, washed and oven dried to record the total seedling biomass as a measure of seedling vigor.

### Statistical analysis

The experiment was a split-plot randomized complete block design. Temperature was the main plot factor and the sub-plot factor was the genotype. Replicated observations for each trait were analyzed for means and standard error and ANOVA was performed using R v.3.6.1^35^.

## Results

### Environmental results

#### Implementation and distribution of HNT stress

**Figure 3:**
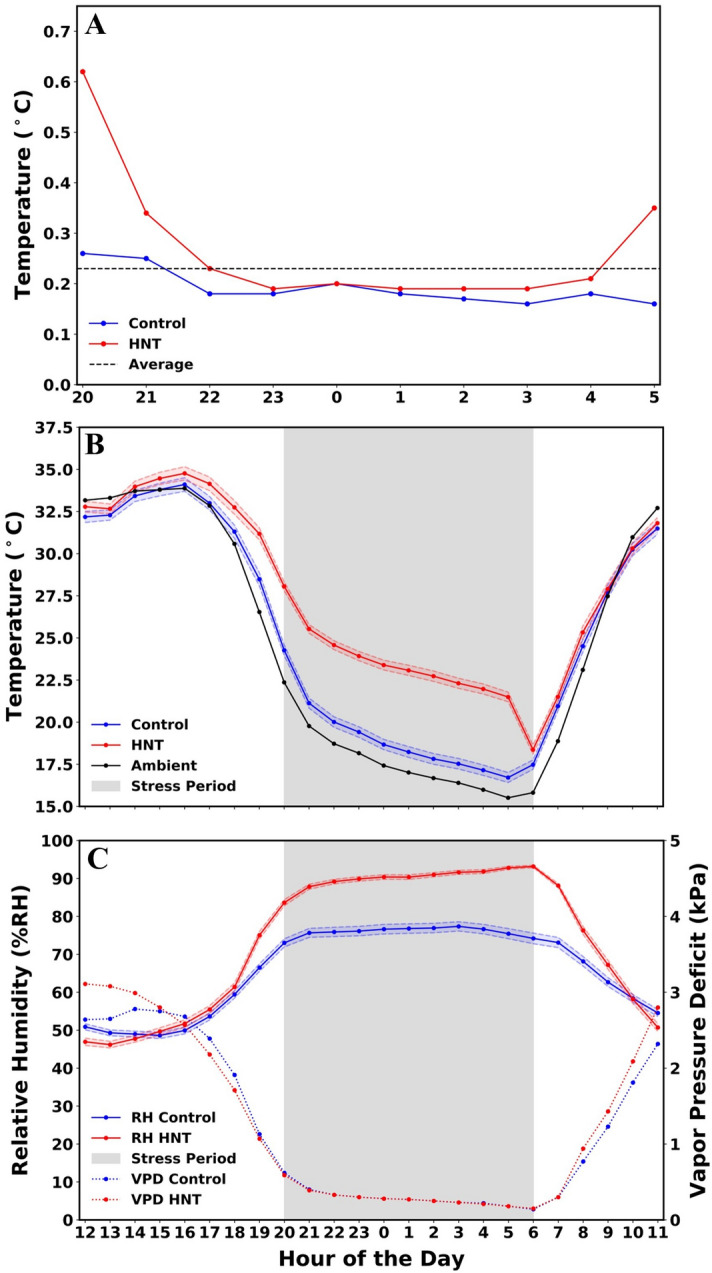
Environmental conditions in control and stress tents. (**A**). Comparison of the temperature differences between the HOBO data loggers within the same tents for control, HNT and overall average. The lower the temperature on the graph represents a more uniform heat distribution as the sensors were spread randomly throughout each tent, see Fig. 2. (**B**). A comparison of the average temperature within the stress tents, control tents and ambient conditions starting at 12:00 PM and ending at 12:00 AM over the entire duration of the experiment. (**C**). The control and stress tents average relative humidity and vapor pressure deficit are shown throughout the day. 95% confident intervals are represented by the shaded regions above and below the control and stress lines for temperature (**B**) and relative humidity (**C**).

Through the use of horizontal air flow fans and convection tubing in each tent, a uniform distribution of heat was achieved (Fig. 3A). On average, the system measured a 0.6 °C differential between the six temperature sensors within the stress tents (Supplementary Table 3). The HOBO UA 002-64 Pendent data loggers were also used to validate uniform heat distribution in control and stress tents. The HOBO Pendent loggers measured an average of 0.2 °C temperature difference at any given time within the control and stress tents (Fig. 3A).

The varieties within the stress tents were exposed to an elevated HNT conditions which averaged + 3.8 °C, as measured by the Raspberry Pi sensor array, compared to the average night-time temperature of the control tents (Fig. 3B). The overall average temperature during the stress period, in the stress tents was 22.1 °C while it was 18.4 °C in the control tents (Supplementary Table 3). The temperature within the stress tents and the control tents began to diverge from 6 PM as the closing of the stress tents was initiated (Fig. 3B), reaching + 2.8 °C and + 3.5 °C by 7 and 8 PM, respectively. The average differential continued to rise and maintained at ~ 3.8 °C from 10 PM until returning to ambient conditions, as the experiment was turned off and tents were opened at 6 AM.

#### Effective day time ambient temperature

Between 7 AM and 6 PM, the control and heat tents were on average 0.4 °C warmer than the ambient conditions as measured by the HOBO UA 002-64 Pendent data loggers (Fig. 3B). The tents had the largest differential away from ambient in the early morning immediately after the HNT stress was released, which were 2.4 °C warmer than ambient. This was reduced to 1.9 °C by 8 AM and the difference was only 0.4 °C at 9 AM. Between 10 AM and 1 PM the tents temperature average was cooler than the ambient conditions and after 1 PM they stayed within 1 °C of ambient conditions until 6 PM when steps to cover the tents were initiated (Fig. 3B).

#### Relative humidity, vapor pressure deficit and carbon dioxide

During the non-stress period the stress tents relative humidity (RH) was on average 2.3% higher than the control tents (Fig. 3C). From 10 AM to 6 PM the difference in RH between the two sets of tents ranged between 0.2 and 4%. During this period, the control tents had a slightly higher RH than the stress tents for the majority of this period, with an average of 52.6% and 52.0% respectively. The vapor pressure deficit (VPD) within the tents during the non-stress period ranged from 0 to 0.5 kPa but the overall average difference was only 0.1 kPa (Fig. 3C). During the stress period, the stress tents RH ranged from 11 to 19% higher than the control tents (Fig. 3C), with 0.01 kPa lower VPD in the stress tents compared to the control tents. The overall average CO_2_ concentration within the stress tents was 538 ppm but ranged from 457 ppm when the tents were first closed to 565 ppm while the CO_2_ concentration within the control tents was 544 ppm and ranged from 480 to 576 ppm (Supplementary Table 3).

### Agronomic responses to HNT stress

#### Everest check lines

The results from each Everest check line were grouped based on their position within the tent (blocks one through four and blocks five through eight) and ANOVA was performed to evaluate the differences within each tent based on grain yield (g/m^2^), 200 kernel weight (g) and harvest index. At a 95% confidence interval, there were no significant differences between the two different groupings within each of the tents for all three traits (Supplementary Table 4). This further reinforced the conclusion of a highly consistent and uniform distribution of heat stress within each of the heat tents, supporting the findings presented in Fig. 3.

**Figure 4:**
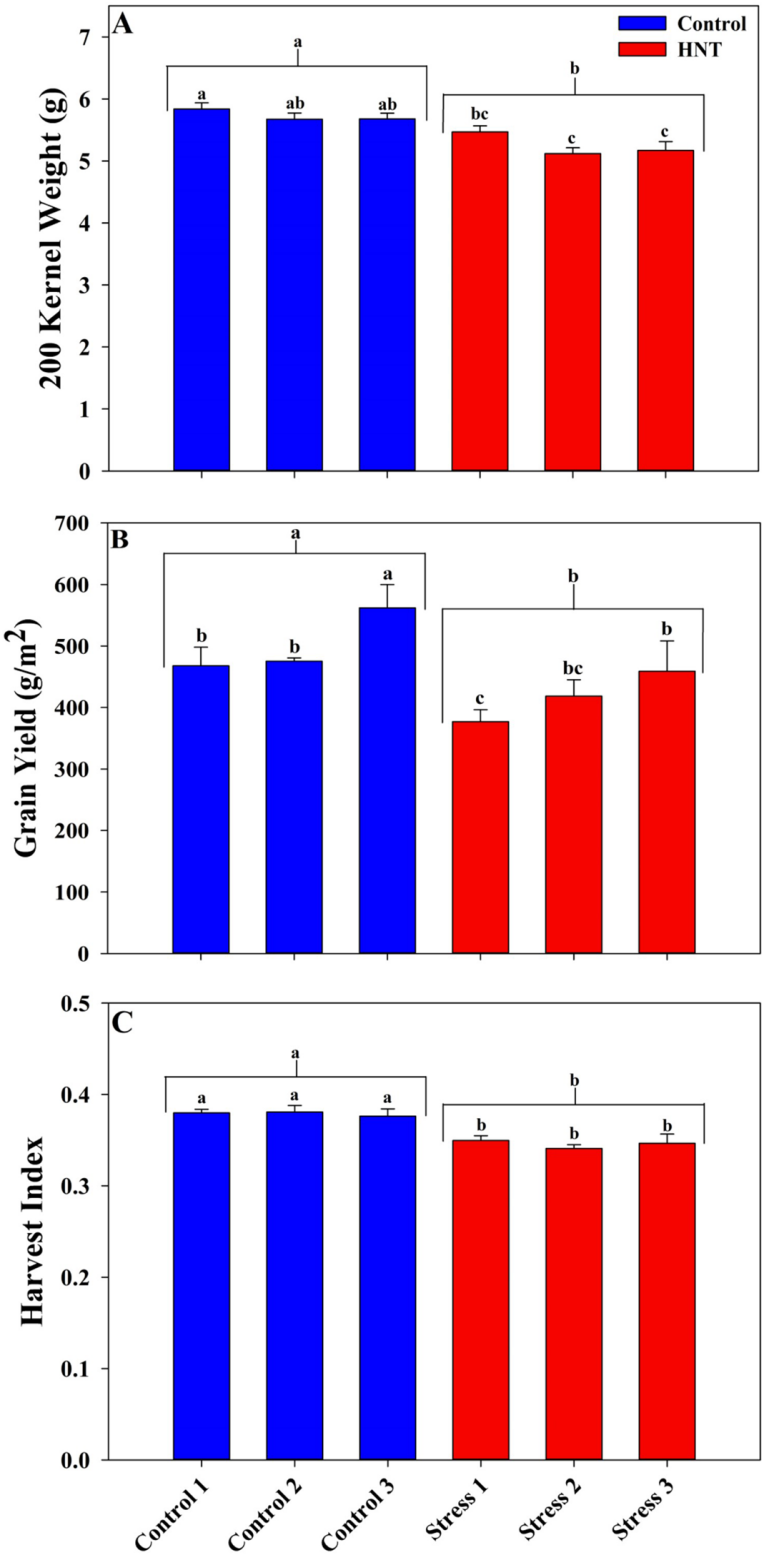
Comparison of Everest check lines planted randomly in each of the eight blocks within a tent. Comparison of 200 Kernel Weight (g) (**A**), grain yield (g/m^2^) (**B**) and Harvest index (**C**) in the three control and stress tents. Each column is an average of eight rows of check line Everest and bars indicate ± SE. Letters above the bars indicate groups differing significantly between individual stress repetitions and the letters above the brackets indicate groups differing significantly between overall average between control and stress groups (p < 0.05).

Across the tents, there was no significant difference between the control tents for both 200 kernel weight and harvest index (Fig. 4A and C). However, with grain yield, both control tents 1 and 2 did not differ significantly, but control tent 3 recorded a significantly higher grain yield compared to control tents 1 and 2 (Fig. 4B). This can be attributed to the placement of control tent 3 towards the southern end of the plot, which was possibly influenced by the inputs from the previous sorghum crop. This was also reflected in the heat stress tent 3, which was paired with control tent 3 (Fig. 4B) (Supplementary Fig. 6). Based on the design of the system wherein one control tent was paired with one stress tent, comparing findings between the pairs is appropriate while determining the effectiveness of the HNT stress (Supplementary Fig. 6). Comparing 200 kernel weight and harvest index between the respective control and heat tents, recorded a statistically significant difference (Fig. 4A and C). Although there was a significant difference between two tent pairs for grain yield, overall the HNT resulted in a significant reduction in 200 kernel weight, grain yield and harvest index compared to control (Fig. 4).

#### Grain-filling duration

**Figure 5:**
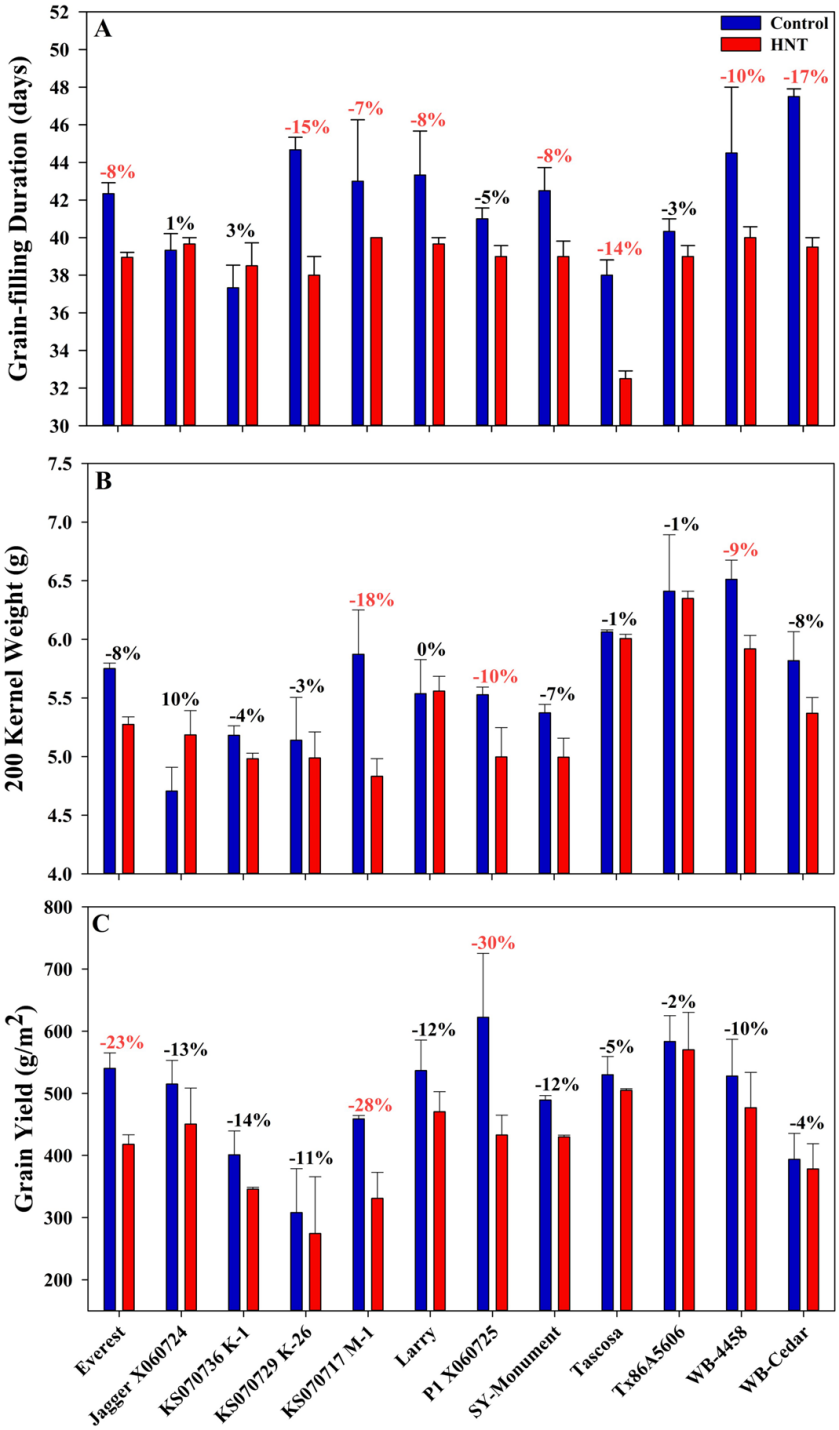
Agronomic response of wheat genotypes exposed to HNT stress during grain-filling. Grain-filling duration (**A**), 200 kernel weight (**B**) and grain yield (**C**) of 12 winter wheat genotypes exposed to HNT stress and control conditions for the entire grain-filling period. Reductions in red signify significant reduction (p < 0.05).

Recording the start of flowering and physiological maturity for all lines across both treatments allowed for the determination of the grain-filling duration. HNT stress had a significant effect with treatment, genotype and their interaction on grain-filling duration (Fig. 5A; Table 2). Exposure to HNT stress reduced grain-filling duration in 10 of the 12 genotypes (except KS 070736K-1 and Jagger X060724), with eight of the 10 recording a significant reduction (Fig. 5A). Averaged across the genotypes, grain-filling duration was reduced by 3.33 days or 7.7% or 2.0% per °C. The largest reduction of 5.5 days was recorded in the genotype Tascosa, while the least affected variety was KS 070736K-1 wherein the grain-filling duration increased by over a day (Fig. 5A).

**Table 2:** Probability values of effects of temperature (T), genotype (G) and T × G interaction on biomass, grain yield and quality traits.

Traits	Variables			Main effect of temperature (mean)	
	T	G	T × G	Control	HNT
Grain-filling duration (d)	0.004	< 0.001	0.031	42.0^a^	38.9^b^
200 kernel weight (g)	< 0.001	< 0.001	0.012	5.7^a^	5.3^b^
Grain yield (g m^-2^)	0.002	< 0.001	0.729	512.8^a^	424.9^b^
Seed number (m^-2^)	0.012	< 0.001	0.874	18,112.6^a^	15,879.8^b^
Biomass (g m^-2^)	0.11	< 0.001	0.649	607.7^a^	570.0^a^
Harvest index	0.013	< 0.001	0.122	0.38^a^	0.35^b^
Starch concentration (%)	< 0.001	< 0.001	0.6149	59.0^a^	50.0^b^
Protein concentration (%)	0.431	< 0.001	0.932	14.1^a^	14.5^a^

Means were separated using Tukey’s honest significant difference (HSD) test at p = 0.05 and statistically significant differences (p < 0.05) between control and HNT effects are indicated by superscripts.

#### 200 Kernel weight and grain yield

The effect of HNT stress on 200 kernel weight was significant at the treatment, genotype and their interaction levels (Fig. 5B; Table 2). HNT stress reduced the overall 200 kernel weight of the tested genotypes by an average of 4.8% or 1.3% per °C. Of the 12 genotypes, 10 recorded a reduction in 200 kernel weight. The largest reduction in 200 kernel weight was observed in KS 070717M-1 followed by P1 X060725 and WB-4458 and, conversely, a substantial increase was recorded in Jagger X060724.

All 12 varieties tested had a lower grain yield with HNT exposure compared to control conditions. P1 X060725 recorded the largest reduction in grain yield with a 30.4% or 189.29 g/m^2^ reduction as compared to control conditions. The variety with the lowest reduction was TX86A5606 and the reduction in grain yield averaged 13.6% or 68.60 g/m^2^ or 3.6% per °C across all 12 genotypes. Treatment and genotype had a significant impact but not their interaction (Fig. 5C and Table 2). Everest, KS 070717M-1 and P1 X060725 recorded a significant reduction in grain yield.

Comparing the grain-filling duration and yield changes due to HNT stress can help elucidate genotypic variations in response to stress. WB-Cedar and Tascosa had two of the largest reductions in grain-filling duration (− 16.8% and − 14.5%, respectively), however they also reported two of the lowest reductions in yield at − 3.9% and − 4.7%, respectively (Fig. 5). Alternatively, KS 070717K-1 and Jagger X060724 had increased grain-filling durations (3.1% and 0.8%, respectively), and responded with substantial reductions in yield of − 13.8% and − 12.5%, respectively. This comparison shows that, even though the grain-filling period was reduced, WB-Cedar and Tascosa were able to overcome this reduction and yielded close to non-stress conditions. While KS 070717K-1 and Jagger X060724 were able to maintain their grain-filling duration but not grain yield under stress. This indicated that there could be other physiological processes that lead to yield reduction under HNT, besides reduced grain-filling duration.

#### Seed number, aboveground biomass and harvest index (HI)

Seed number was significantly affected by the treatment and genotype but not their interaction (Supplementary Fig. 7A; Table 2). The average reduction in seed number was 9.0% or 2.4% per °C with the highest reduction found in P1 X060725 followed by Jagger X060724 while WB-Cedar had a marked increase in seed number. Eleven of the 12 genotypes had lower seed number, while the reductions in P1 X060725 and Jagger X060724 were significant.

Aboveground biomass varied significantly with genotype but not with treatment and their interaction (Table 2). The two genotypes with the largest reduction were P1 X060725 and Tascosa. On average, the 12 genotypes recorded a 5.4% or 1.4% per °C reduction in the biomass. Tx86A5606 has the largest increase in aboveground biomass at 17%, which is due to higher variation in measurements ranging from a 3.5% reduction to a 50% increase in biomass (Supplementary Fig. 7B). HNT stress had a significant effect on HI with treatment and genotype but not with their interaction (Table 2; Supplementary Fig. 7C). While eight genotypes recorded a reduction in HI, the only genotype that was significantly reduced was P1 X060725 (Supplementary Fig. 7C).

#### Starch and protein concentration

The HNT stress effect on grain starch concentration was statistically significant at the treatment and the genotype level but not their interaction (Table 2). Eleven of the 12 genotypes had reduced starch concentration, with just KS 070736K-1 not affected negatively (Table 3). The largest reduction was in KS 070717M-1 with the starch concentration reducing from 56.1% under control conditions to 37.2% with stress. The only other genotype to record a significant reduction in starch concentration was Tascosa. On average, the reduction in starch concentration among the 12 genotypes was 15.3% (Table 3). The average protein concentration of the 12 genotypes increased by 2.9% with SY-Monument having the largest increase. Of the 12 genotypes only three recorded lower protein concentration, and not varying significantly with treatment, genotype and treatment interaction (Table 3).

**Table 3:** Starch and protein concentration (%) of mature seeds in 12 field-grown winter wheat genotypes exposed to HNT and control environments during grain filling.

Genotype	Starch concentration (%)			Protein concentration (%)		
	Control	HNT	% difference	Control	HNT	% difference
Everest	59.80	46.87	− 21.62	14.92	15.69	5.22
Jagger X060724	59.27	48.17	− 18.73	14.55	14.57	0.13
KS 070736K-1	48.82	51.80	6.12	14.27	14.73	3.20
KS 070729K-26	54.33	44.34	− 18.40	13.74	14.42	4.98
KS 070717M-1	56.07	37.20	− 33.67	15.68	15.56	− 0.73
Larry	49.27	45.31	− 8.04	14.46	14.25	− 1.45
P1 X060725	48.36	39.99	− 17.32	14.36	14.99	4.37
SY-monument	63.23	59.40	− 6.06	13.13	14.02	6.80
Tascosa	67.77	51.15	− 24.52	12.98	13.28	2.34
Tx86A5606	59.66	51.02	− 14.47	12.64	13.21	4.51
WB 4458	63.49	59.98	− 5.52	13.36	14.27	6.83
WB-cedar	78.29	64.66	− 17.41	14.95	14.92	− 0.25
Overall average	59.03^a^	49.99^b^	− 15.31	14.09^a^	14.49^a^	2.89

Means were separated using Tukey’s honest significant difference (HSD) test at p = 0.05 and statistically significant differences (p < 0.05) between the overall average of control and HNT are indicated by superscripts.

#### Seedling emergence and vigor

Both emergence index and emergence percentage did not vary significantly in the controlled environment growth chamber experiment, in seeds obtained from HNT experiment in the field. On average, the emergence index was increased by 5.6% and the total emergence percentage was reduced by 5.3%. Total seedling biomass was significantly affected by the HNT treatment, with an average reduction of 6.94% or 3.9 g recorded across the genotypes. Among the genotypes, Jagger X060724 and P1 X060725 recorded a significant reduction in seedling biomass (Supplementary Table 5).

## Discussion

### Scalability and effectiveness in imposing HNT stress on a large scale

The major challenge faced with scaling up the HNT stress imposing prototype system to a custom-built large scale field-based infrastructure was a significantly updated cyber-physical system which could successfully impose and measure a predetermined temperature differential^30^. In addition, this had to be achieved without significantly altering the environmental conditions within the tents during the day compared to the outside temperature and implement HNT stress uniformly throughout the tent for the entire duration of the grain-filling period. The improved system achieved an average stress of + 3.8 °C, an improvement over the prototype, which was able to achieve + 3.2 °C differential^30^, with a target of + 4.0 °C in both cases. This increase of 3.8 °C is similar to the results obtained in other field-based HNT experiments utilizing much smaller enclosures, wherein a single genotype was tested^27,28^. Hence, the presented system demonstrates for the first time the possibility of imposing HNT stress consistently on a large scale with higher precision than the previous prototype or other small scale enclosures.

Unlike Hein et al.^30^, the improved system included a full-fledged ventilation to exhaust the off-gasses from the combustion of propane, similar to many greenhouse structures used for horticultural purposes^36^. Despite this, a higher CO_2_ concentration in both control and stress tents (Supplementary Table 3) reflected an increase in night respiration, altering the carbon balance^28^. A similar phenomenon was demonstrated in wheat grown under controlled environment conditions, wherein HNT resulted in increased carbon loss due to high night respiration, leading to lower grain yield^23,24^. A similar response has been captured in rice grown in chambers and field conditions^10,12^. Although the study was unable to estimate the impact of HNT on night respiration on individual accessions, the tent-scale increase in CO_2_ concentration provides justification for loss of carbon under HNT affecting yield and quality in field grown wheat (Table 3 and Fig. 5). However, a larger increase in CO_2_ levels with HNT could not be captured due to the structural settings of the tents that facilitated air exchange from outside the tents, although minimal, to maintain comparable RH with the control tents.

Another challenge with the upscaled experimental design was not only to impose stress on a much larger area but also to ensure that the HNT stress was applied uniformly on a single row layout. This was demonstrated using actual temperature sensor array from the cyber-physical system which measured an average difference of 0.6 °C between the six sensors spread randomly throughout the tent. This was further validated independently from two HOBO pendent loggers which measured an average difference of 0.2 °C, across tents for the entire stress period. Further, the imposition of temperature stress per se may not completely justify the uniformity of the system unless a similar measure is observed at the plant level. The uniformity in stress imposition was demonstrated in the statistical similarity in yield and yield components in the check line, Everest, which was planted randomly in each of the eight blocks in all six tents (Fig. 4; Supplementary Table 4). In summary, a combination of + 3.8 °C average heat stress with a very small average differential between 0.2 and 0.6 °C between sensors within a tent, a non-significant variation with yield and its components in a common check line confirms that the design was able to successfully impose HNT stress both consistently and uniformly throughout the grain-filling duration. In addition, the sophistication added to the physical components, revised algorithms and improvements to the cyber-physical system allowed the methodology to be successfully scaled up to impose HNT stress on a large diversity panel.

### Comparative response of HNT across chambers and field based facilities

**Figure 6:**
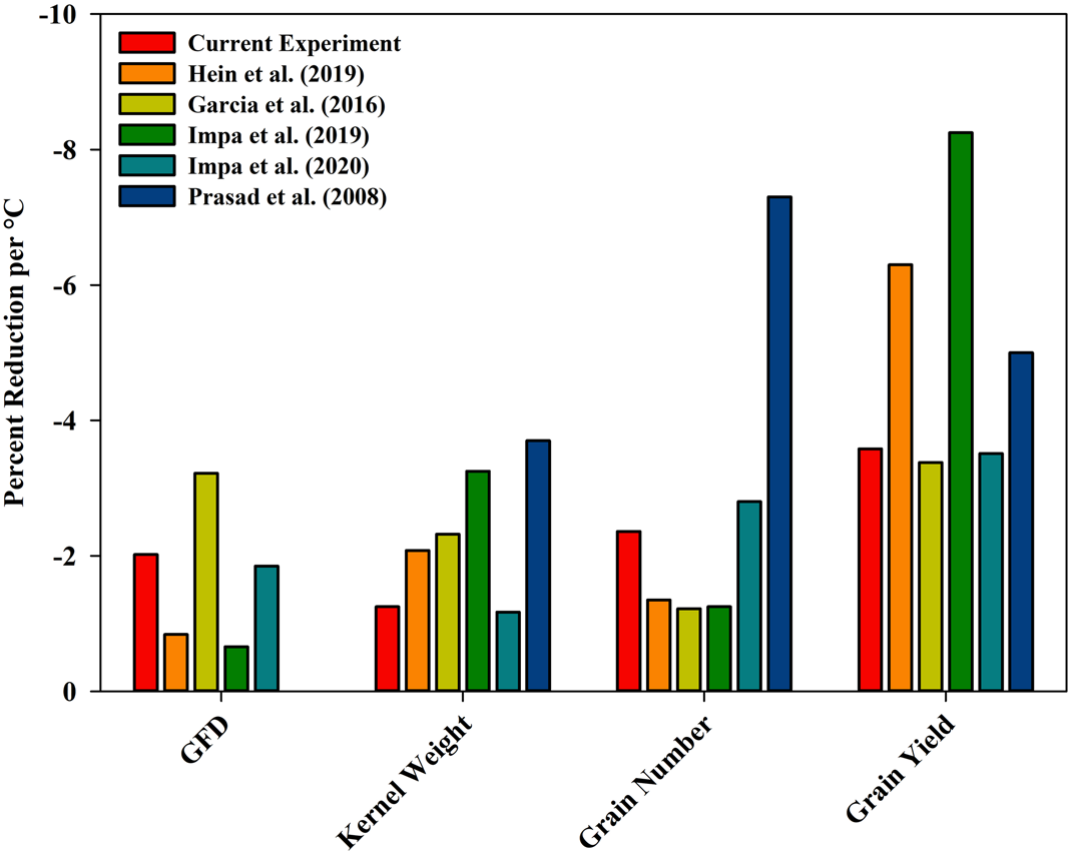
Graphical comparison of HNT stress impact on key agronomic parameters between six independent experiments in wheat. The results are presented as percent reduction per °C of heat stress and represents the average of all genotypes within the experiment. If multiple HNT stress levels were tested in a single experiment, the average reduction was calculated for each treatment level and then the results of each treatment were averaged to gain an overall experimental percent reduction. In the current experiment HNT stress was imposed post-anthesis through maturity with an average stress level of + 3.8 °C and control of 18 °C. In Hein et al.^30^ (field-based) HNT stress was imposed post-anthesis through maturity with an average stress level of + 3.2 °C with a 22 °C control. Both Hein et al.^30^ and the current study had 12 winter wheat genotypes. In Garcia et al.^28^ (field-based) HNT stress was imposed post-anthesis through maturity with an average stress level of + 4.15 °C during two different years (+ 4.9 °C with a 17 °C control and + 3.4 °C with a 14.3 °C control) with a single genotype of winter wheat. In Impa et al.^23^ (controlled environment growth chamber) HNT stress imposition after heading and maintained a + 8 °C HNT stress through maturity with a 15 °C control, using six different genotypes. In Impa et al.^24^ (controlled environment growth chamber) HNT stress was applied post-anthesis through maturity. The experiment had five levels of heat stress (+ 3, + 6, + 8, + 10, and + 12 °C) and a 15 °C control and 10 different genotypes. In Prasad et al.^26^ (controlled environment growth chamber) HNT stress applied at the booting stage until maturity. The experiment had three levels of heat stress (+ 3, + 6, and + 9 °C) with a 14 °C control and utilized two spring wheat cultivars.

HNT beyond 23 °C starting from booting until maturity had a significant impact on grain-filling duration in wheat grown under chamber conditions^24^. The response to HNT across scales was consistent, wherein the grain-filling duration was reduced by 3 days in chambers at 20 °C and the current field study at 22 °C^28^ (Figs. 5 and 6). The reduction in grain-filling duration caused by early senescence in winter wheat due to HNT stress is one of the main factors responsible for lower 200 kernel weight. Reduced grain-filling duration on exposure to HNT lowers the active photosynthetic area and duration affecting the overall assimilate accumulation and supply to the developing grains, inducing yield and quality losses^23,24^ (Table 3, Fig. 5).

HNT exposure in the current study, on average, reduced the 200 kernel weight by 4.8% or 1.3% per °C, which was more consistent with other field-based experiments as compared to results from controlled environment chamber studies^28,30^ (Fig. 6). Grain number was reduced on average by 9.0% or 2.4% per °C in the current study with post-flowering HNT exposure. HNT stress exposed from flowering until maturity induced a higher reduction in seed number under controlled environment study^24^. A higher reduction in grain number was attributed to the impact of HNT on the later developing tillers, with the sensitive reproductive organ development i.e., gametogenesis coinciding with the stress period^24,32,37^. Similarly, a longer duration of HNT stress starting from booting until maturity would have impacted the later developing tillers more significantly, leading to a much higher reduction in grain numbers^26^. The combination of reduced grain-filling duration (2.0% per °C), 200 kernel weight (1.3% per °C) and grain number (2.4% per °C) caused a significant yield reduction of 3.6% per °C of HNT stress (Fig. 6). Recent field experiments recorded a 3.4% and 6.3% per °C reduction in grain yield under post-flowering HNT stress^28,30^, while growth chamber experiments reduced grain yield by 3.5% and 5% per °C^24,26^ (Fig. 6). Overall, the impact of HNT across scales was consistent with yield and its components and the deviations seen in some cases can be attributed to the range in genetic diversity in the respective study and the intensity and duration in night temperatures.

The reduction in grain yield in this experiment was lower than previously reported with 3.2 °C higher night temperature^30^, which can be attributed to the inter-annual ambient night temperature variations. This is apparent when comparing this study to Hein et al.^30^, which had a slightly lower heat stress increase (+ 3.2 °C vs + 3.8 °C) but saw a much larger reduction in yield per degree Celsius (6.3% vs 3.6%). This is due to the ambient conditions during the experimental period being warmer in Hein et al. compared to the current experiment^30^. The average temperature of stress induced in Hein et al. was 26 °C while the average temperature in the current study was 22 °C which accounts for the differing degrees of impact induced by HNT stress^30^.

Elevated night temperatures negatively affect grain quality by altering the major constituents of the grain. The major impact observed in this experiment was HNT stress induced reduction in total starch concentration (Table 3), which is in line with a recent growth chamber study^24^. A significant reduction in grain starch due to HNT allowed for additional protein and lipid accumulation in two contrasting genotypes. This study found a significant increase in protein concentration in KS 070717M-1 but did not observe a similar increase in the tolerant SY-Monument^24^. A similar striking response with protein concentration was not observed in our study but the response in starch reduction between the susceptible KS 070717M-1 and the tolerant SY Monument was in agreement with Impa et al.^24^. Though starch and protein deposition in grains is initiated at the same time, starch deposition is completed around 45 days after flowering. Protein deposition, however, reaches its peak at around 20 days after flowering and hence is not equally affected due to shortened grain-filling duration^38,39^. Findings from the current field study possibly captures this phenomenon more accurately due to the rapid rate of terminal senescence compared to significantly slower senescence rate under well-watered conditions maintained until maturity under chamber conditions^24^. In summary, it can be hypothesized that a significant loss in grain starch due to HNT under field conditions may not always result in increase in grain protein.

## Conclusion

The methodology first proposed in Hein et al.^30^ was successfully upscaled through significant upgrades in the heat tent structure, heating system, and a fully redesigned cyber-physical system. The large mobile field-based infrastructure was successful in imposing HNT stress uniformly within the tent and consistently throughout the grain-filling duration (Table 1). These comparable results in both agronomic and quality parameters from growth chambers, small field-based enclosures and large field-based experiments reveal consistent effects of HNT in a variety of testing environments. Having demonstrated the agreement of findings across controlled environments and field conditions, provides new avenues to use high throughput phenotyping indices identified under chamber conditions^14^. Extending chamber based indices will facilitate effective utilization of advances in phenotyping to large scale field infrastructure involving diversity panels and mapping populations. In addition, the confidence provided with these comparative assessments across scales strengthen our ability to take relevant decisions on contrasting genotypes, traits, physiological and molecular markers for enhancing HNT tolerance in wheat and other field crops.

## Supplementary Information

Supplementary Legends.

Supplementary File 1.

Supplementary Figure 1.

Supplementary Figure 2.

Supplementary Figure 3.

Supplementary Figure 4.

Supplementary Figure 5.

Supplementary Figure 6.

Supplementary Figure 7.

Supplementary Table 1.

Supplementary Table 2.

Supplementary Table 3.

Supplementary Table 4.

Supplementary Table 5.

## Data Availability

Custom Python Thermostat Controller Code can be found at https://github.com/danwwagner/thermostat-controllers/tree/v2.0.0 or https://zenodo.org/record/3332925#.XqSNQchKiUk. All other datasets used and/or analyzed during the current study are available from the corresponding author on reasonable request.

## Abbreviations

HNT: High night temperature
BTU: British thermal unit
FPM: Feet per minute
RTC: Real-time clock
CFM: Cubic feet per minute
