# Supplementary material for: Improved cyber-physical system captured post-flowering high night temperature impact on yield and quality of field grown wheat
Source: Sci Rep. 2020 Dec 17;10:22213. doi: 10.1038/s41598-020-79179-0 (PMC7747627; doi:10.1038/s41598-020-79179-0)
Supplement: Supplementary file 1 — Supplementary Legends. [file 41598_2020_79179_MOESM1_ESM.docx]

**Supplementary Information**

**Supplementary Fig. 1.** An illustration detailing individual components of the mobile field-based infrastructure. (A). Overhead view of field layout depicting the control tents in their night setting and the stress tents in day setting. Also visible is the roof roll-up ventilation and the sidewall roll-up ventilation systems. (B). Overhead view of stress tent with circulation fan, roof roll-up ventilation system and exterior propane tank. (C). Interior view of stress tent in its night setting looking towards the propane heater. Circulation fan, propane heater, and Raspberry Pi enclosure are visible. (D). Interior view of stress tent in its night setting looking opposite of the propane heater with the propane heater and convection tubing visible centrally located running the entire length of the stress tent.

**Supplementary Fig. 2.** The Raspberry Pi system for a control tent, stress tent, and its enclosure. (A). A visual display of the enclosure for a Raspberry Pi system for a control tent. Enclosed is the Raspberry Pi itself, a DS32131 Real Time Clock and the connections between the Raspberry Pi and the six temperature sensor array. (B). The interior of an enclosure for a stress tent consisted of a Raspberry Pi, the four relays used to control the propane heater, the connections to the six temperature sensor array, and a DS32131 Real Time Clock. (C). Exterior view of a Raspberry Pi enclosure with the six temperature sensor array coiled in preparation for installation. The enclosure and array were installed as seen and then the sensors uncoiled along the roof trusses into their predetermined positions (see Fig. 2).

**Supplementary Fig. 3.** The set of four relays which were used to control the propane heater. (A). Description of each of the three relays used and the unused relay indicated. The relays' NO ports were coalesced and connected to the 24VAC line off the heater, and the COM ports were connected to the G, W1, and W2 ports of the heater. (B). The relay board supplied with power as indicated by the Board Power light and the relays engaged indicated by the light next to each relay. This is the state of the relays to turn on stage 2 heating when the temperature differential was below the desired value for heat stress. The propane heater was able to function on three different settings; fan only (no heat), stage 1 heating (50%), and stage 2 heating (100%). After initial attempts to utilize both stage 1 heating and stage 2 heating, stage 1 heating was found to be insufficient to make a significant change within the tent and only stage 2 was used.

**Supplementary Fig. 4.** A system wiring diagram that connected the Raspberry Pi to the relays for the stress tents and to the temperature sensors, RTC module, and CO2 sensor for all tents. The Raspberry Pi can be seen in Supplementary Fig. 2 and the relays are seen in Supplementary Fig. 2 and 3. The Raspberry Pis were placed in both the control and stress tents and a diagram of their interactions can be seen in Fig. 2.

**Supplementary Fig. 5.** Two methods were compared to quantify the yield and yield components to ascertain the impact of Fusarium Head Blight. The seeds were separated into infected and non-infected categories and the agronomic parameters were extrapolated from the non-infected seed category. These results were then compared to the raw data results, which showed extremely high correlation between both methods and the non-categorized whole sample results were considered for all further analysis. Graphs A, B and C show the control data comparison for 200 kernel weight, grain yield and harvest index, respectively. Graphs D, E, and F show the HNT comparison for the 200 kernel weight, grain yield, and harvest index. While graphs G, H and I show the percent differential comparison for 200 kernel weight, grain yield, and harvest index, which compared the change in percentage between control and HNT.

**Supplementary Fig. 6.** Diagram of field layout showing tents designated as control and stress tents. The field layout also shows the pairing of control and stress tents for the purposes of communication between the Raspberry Pi’s between tents.

**Supplementary Fig. 7.** Seed number (A), biomass (B) and harvest index (C) in 12 winter wheat genotypes exposed to HNT stress and control conditions during the entire grain-filling period. Reductions in red signify a statistically significant reduction (p <0.05).

**Supplementary Table 1.** Field-based infrastructure components.

**Supplementary Table 2.** Raspberry Pi cyber-physical system components.

**Supplementary Table 3.** Experiment Environmental Conditions. Hourly average environmental conditions over the duration of the experiment. N = Number of Observations

**Supplementary Table 4.** Check line Everest variance for agronomic traits.

**Supplementary Table 5.** Next generation seedling vigor experimental results.

**Supplementary File 1.** Cyber-physical system details and code.
