# Supplementary material for: Improved cyber-physical system captured post-flowering high night temperature impact on yield and quality of field grown wheat
Source: Sci Rep. 2020 Dec 17;10:22213. doi: 10.1038/s41598-020-79179-0 (PMC7747627; doi:10.1038/s41598-020-79179-0)
Supplement: Supplementary file 2 — Supplementary File 1. [file 41598_2020_79179_MOESM2_ESM.docx]

Improved cyber-physical system captured post-flowering high night temperature impact on yield and quality of field grown wheat

**Running Title –** HNT affects field-grown wheat yield & quality

**Authors** – Nathan T. Hein^1^, Raju Bheemanahalli^1^, Dan Wagner^2^, Amaranatha R. Vennapusa^1^, Carlos Bustamante^1^, Troy Ostmeyer^1^, Meghnath Pokharel^1^, Anuj Chiluwal^1,4^, Jianming Fu^1^, Dhanush S. Srikanthan^1^, Mitchell L. Neilsen^2^, S.V. Krishna Jagadish^1^*

**Affiliations**

^1^Department of Agronomy, Kansas State University, Manhattan, KS 66506, USA

^2^Department of Computer Science, Kansas State University, Manhattan, KS 66506, USA

**Current Address**

^4^Department of Plant and Soil Sciences, University of Kentucky, Lexington, KY 40546, USA

**Emails** - [Nhein@ksu.edu](mailto:Nhein@ksu.edu), [Rajubr@ksu.edu](mailto:Rajubr@ksu.edu), [Danwagner@ksu.edu](mailto:Danwagner@ksu.edu), [amarv@ksu.edu](mailto:amarv@ksu.edu), [Carlos1@ksu.edu](mailto:Carlos1@ksu.edu), [tjostmeyer@ksu.edu](mailto:tjostmeyer@ksu.edu), [pokharelmeg@ksu.edu](mailto:pokharelmeg@ksu.edu), [anujchiluwal@gmail.com](mailto:anujchiluwal@gmail.com), [dhanush.srikanth28@gmail.com](mailto:dhanush.srikanth28@gmail.com), [jianming@ksu.edu](mailto:jianming@ksu.edu), [neilsen@ksu.edu](mailto:neilsen@ksu.edu), [kjagadish@ksu.edu](mailto:kjagadish@ksu.edu)

Hardware Components and Connections

The relays required five wires to run: 5V power, ground, and an input signal for each relay; when raised high high, this signal completed the heater circuit on the control board and facilitated the heater’s operation. These wires were connected to the Pi on the following pins using the board’s physical numbering scheme (Fig. 6):

- Pin 4 provided 5V power.
- Pin 9 provided ground.
- Pin 11 provided the first relay’s input signal.
- Pin 15 provided the second relay’s input signal.
- Pin 13 provided the third relay’s input signal.

Each of the MCP9808s and the RTC required four lines: 5V power, ground, data (SDA), and clock (SCL). All six MCP9808 and the RTC were wired in parallel; the MCP9808s were interfaced with individually by connecting a combination of three address pins to 5V power. The MCP9808s were placed throughout each tent by using 18-gauge, 4 conductor sprinkler wire for waterproofing. Each sensor’s wires were consolidated, and then connected to the physical pins on the Pi:

- Pin 2 provided 5V power.
- Pin 6 provided ground.
- Pin 3 provided the SDA signal.
- Pin 5 provided the SCL signal.

The relays were connected to the heater’s internal control board via the normally open (NO) and common (COM) ports (Supplementary Figure 3). Each NO port was connected to the heater’s 24VAC line as the COM ports were grounded in the relays. The COM ports were used for each of the signal lines: call for fan (G), stage one heat (W1), and stage two heat (W2); first stage heat required both G and W1 to be connected to 24VAC while second stage heat required all three signal lines to be connected to 24VAC.

The MH-Z19 sensor also required four lines: 5V power, ground, transmit (TX), and receive (RX). The sensor ran on the Universal Asynchronous Receiver/Transmitter (UART) protocol and connected to the physical pins on the Pi like below (Fig. 6)[40]:

- Pin 2 provided 5V power.
- Pin 6 provided ground.
- Pin 8 provided the RX signal.
- Pin 10 provided the TX signal.

Software Description

The code was written in Python version 2.7 due to its simplicity, familiarity, and availability of software libraries for the system’s sensors.[41,42,43] The software exists as four scripts: main.py, sensor.py, heatcontroller.py, and controlcontroller.py; the latter two are for heat or control tents, respectively. When the Pi received power, it booted up and immediately began initializing the system. First, the system detected the type of sensor connected *via* the constructor passed into the controller; this is a user-defined interface to the sensor that is employed in their thermostat controller system. Next, it started up the associated controller depending on whether it is located inside a heat or control tent. Finally, the controller initialized the sensor data, system heath logging, reboot counter, and input/output error files for use in the code: these correlate to logging and debugging information used to collect data and handle system faults.

After all system initialization was performed, the main loop was entered. This loop calibrated the CO_2_ sensor by zeroing out the scale. After calibration, it detected all MCP9808s that were connected to the system by polling the I2C bus and recording each unique address; all I2C addresses were, by design, assumed to be for the temperature sensors and must be specified via a list if they are reserved and should not be used for sensor readings. Then, the system attempted to connect to each detected MCP9808 for its temperature reading; if a read error occurred, then the error counter was incremented and compared against the maximum allowable number of errors to determine if a reboot was necessary. If a reboot was required, then the error counter was reset, and the system rebooted to fix the sensor error. However, if the maximum allowable reboots were reached, then the system remained on to maintain the stress period: for control tents, this kept them online for the heat tents to retrieve their outdoor temperature from; for heat tents, this allowed the tent to continue exhibiting heat stress onto the wheat. The number of allowable errors and reboots were recorded to file as a state-saving mechanism to ensure that the system remained in a consistent, controllable state: these were read upon system startup and after reboot. After errors were handled, the connected temperature sensors’ readings were recorded to the sensor data CSV file on the Pi’s storage medium. In the heat tents, the readings were averaged and compared to a 4 °C threshold to determine if the heater should be enabled; it wirelessly retrieved the outdoor temperature from its assigned control tent. If the difference between the indoor and the retrieved outdoor temperatures fell below this threshold, then the system engaged the heater in stage two heat (Fig. 5); stage one heat proved ineffective in maintaining the desired temperature. Meanwhile, the control tents logged their detected temperatures as the outdoor temperature for the heat tents to wirelessly retrieve. Finally, the system slept in a low-power state until the next read interval; by default, this value was set to one minute.

Each step was logged to the system health file for troubleshooting and archival purposes. Any errors that occurred were recorded, as well as the average indoor temperatures for the heat tent and the outdoor retrieved temperature. The main use of this file was to ensure that the system was functioning as intended; if a malfunction occurred, then the file helped isolate and reduce the time required for a technician to solve the problem.

41. Lesson 9: UART [Internet]. 2013 [cited 2020 Apr 25]. Available from: <https://www.simplyembedded.org/tutorials/msp430-uart/>

1. Python Software Foundation. 2019 [cited 2020 Apr 25]. Available from <https://www.python.org>
2. DiCola T. Adafruit python mcp9808 [Internet]. Available from: <https://github.com/adafruit/Adafruit_Python_MCP9808>
3. Ueda T. MH-Z19 [Internet]. Github. 2018 [cited 2020 Apr 25]. Available from: <https://github.com/UedaTakeyuki/mh-z19>

# main.py

from controlcontroller import ControlController

# (1) Change sensor type here

from sensor import MCP9808

# (2) Change sensor type here

# List representation for use if the system will

# contain multiple types of temperature sensors.

# List of reserved i2c addresses

# that are used by components

# other than temperature sensors.

# Make sure that each of the elements

# in this list are hexadecimal strings

# i.e. "1a".

reserved = ["68"]

# You must include a Python implementation that

# uses the Sensor superclass for the

# type of sensor in your system (see sensor.py)

sensor = [MCP9808(reserved)]

# Initialize the controller program

tent_control = ControlController(sensor)

# Enter the main control loop

tent_control.main()

# sensor.py

from abc import ABCMeta, abstractmethod

import subprocess

import Adafruit_MCP9808.MCP9808 as mcp9808

# Include other subclasses for types of sensors to the end of the file

# This position is denoted by another comment

class Sensor(object):

"""

Abstract base class for Sensor objects

"""

__metaclass__ = ABCMeta

@abstractmethod

def __repr__(self):

pass

@abstractmethod

def __init__(self):

pass

@abstractmethod

def num_sensors(self):

pass

@abstractmethod

def detect(self):

pass

@abstractmethod

def read(self):

pass

class MCP9808(Sensor):

"""

Subtype of Sensor class that implements MCP9808 sensor functionality.

"""

def __init__(self, reserved_addr):

self.num_sensors = 0

self.addr_list = []

self.sensor_list = []

self.changed_sensors = False

self.reserved = list(reserved_addr)

def __repr__(self):

return "MCP9808"

def num_sensors(self):

"""

Retrieve the number of sensors detected

"""

return self.num_sensors

def detect(self):

"""

Detects and internally records the number of

MCP9808 connected to the system via i2c.

Initializes each sensor for reading data.

The code naively assumes that each i2c device

is a sensor object; thus, ensure that the class

list reserved has addresses that are used

by i2c devices other than the MCP9808s.

"""

# Command to detect the I2C bus connetions

op = ("sudo i2cdetect -y 1 "

"| sed 's/--//g' | tail -n +2 | "

"sed 's/^.0://g' | sed 's/UU//g'")

# Poll the number of sensors via text manipulation from the I2C bus.

process = subprocess.Popen(op, stdout=subprocess.PIPE, shell=True)

raw_sensors, errors = process.communicate()

# Close the process/file

# In this case, we ignore if it's already terminated/closed.

try:

process.terminate()

except OSError:

pass

# Hexcode of an individual sensor

sensor_name = ''

temp_addr_list = []

# Create the sensor objects.

maxim = len(raw_sensors)

for i in range(0, maxim):

if raw_sensors[i]!= ' ' and raw_sensors[i] != '\n':

sensor_name += raw_sensors[i]

if len(sensor_name) == 2 and sensor_name

not in self.reserved:

# Hexadecimal addresses are two digits long

temp_addr_list.append(sensor_name)

sensor_name = ''

# If a different number of sensors has been detected, update

if (len(temp_addr_list)!= len(self.addr_list)):

for address in self.addr_list:

del address

for sensor in self.sensor_list:

try:

del sensor

except IndexError:

break

del self.addr_list

self.addr_list = list(temp_addr_list)

self.changed_sensors = True

self.num_sensors = len(self.addr_list)

# Begin communication with each sensor

# and add it to the list.

i = 0

for addr in self.addr_list:

if self.changed_sensors:

self.sensor_list.append(mcp9808.MCP9808((int(addr, 16))))

self.sensor_list[i].begin()

i += 1

self.changed_sensors = False

def read(self):

"""

Read sensor data and return the averaged value and each individual

reading in CSV format for logging purposes.

"""

indoor = 0

sensor_readings = ""

for i in range(0, self.num_sensors):

temp = float(self.sensor_list[i].readTempC())

sensor_readings += ("," + repr(temp))

indoor += temp

indoor /= self.num_sensors

return indoor, sensor_readings

# Add other implementations of sensor types here

# heatcontroller.py

#!/usr/bin/env python

# Sensor Reading Author: Adafruit Foundation

# Source: https://bit.ly/1iFB8ZP (DS18B20)

# Adapted and modified by: Dan Wagner

# Agronomy Research, 2018-2019

import logging

import sys

import time

import RPi.GPIO as GPIO

import codecs

import subprocess

import mh_z19

class HeatController:

"""

Controller class that manages the Thermostat system

"""

def __init__(self, sensor_list):

"""

Initializes the controller's variables and list of sensors.

"""

# Designate the type of sensor we are using.

self.sensors = sensor_list

# Keep track of the number of each type of sensors connected.

self.num_sensors = [None] * len(self.sensors)

# Filename for specific tent to write data

self.data_file = '01.txt'

# Format for logging information

self.format = "%(asctime)-15s %(message)s"

# Temperature differential for tent

self.temperature_diff = 4

# Log file interval, in seconds

self.log_interval = 300

# Temperature checking interval, in seconds

self.check_interval = 60

# IP address of the control tent for outdoor temperature monitoring

self.control_ip = '192.168.4.2'

# File location for outdoor temperature from server

# Includes the colon for scp (pi@ip:dir)

self.control_dir = ':/home/pi/thermostat-controllers/src/outdoor .'

# List of sensors connected to the system

self.sensor_list = []

# Initialize the self.indoor temperature

self.indoor = 0

# Initialize the self.delay time period

self.delay = 0

# Initialize self.heater status to OFF

self.heater = "OFF"

# Initialize counter for time elapsed before logging interval

self.cnt = 0

# Set up the relay signal pin

self.signal_pin = 17

# Set up the stage one pin

self.stage_one_pin = 27

# Set up the stage two pin

self.stage_two_pin = 22

# Use the Broadcom SOC channel number

GPIO.setmode(GPIO.BCM)

# Set it as an output pin

GPIO.setup(self.signal_pin, GPIO.OUT)

GPIO.setup(self.stage_one_pin, GPIO.OUT)

GPIO.setup(self.stage_two_pin, GPIO.OUT)

# Pull it low for safety

GPIO.output(self.signal_pin, GPIO.LOW)

GPIO.output(self.stage_one_pin, GPIO.LOW)

GPIO.output(self.stage_two_pin, GPIO.LOW)

# Delimit the next day's individual sensor readings via blank line

self.sensor_readings = codecs.open('sensors.csv', 'a', 'utf-8')

self.sensor_readings.write('\n')

self.sensor_readings.close()

# Instantiate the logging for debugging purposes

self.logger = logging.getLogger("Controller")

# Record number of reboots the system has experienced

self.reboots = ''

# Record the number of recent I/O errors

self.io_errors = ''

# Maximum number of allowable reboots

self.reboot_max = 5

# Maximum number of allowable I2C errors before reboot

self.error_max = 3

# Main loop of the program.

def main(self):

"""

Configure the logger and record the types of

sensors that have been detected by the controller.

"""

self.logger.basicConfig = logging.basicConfig(format=self.format,

filename='control.log',

level=logging.INFO)

self.logger.info('SYSTEM ONLINE')

# Log the types of sensros we have detected in the system

for sen in self.sensors:

self.logger.info('Detected %s sensors', str(sen))

# Calibrate current CO2 to 410ppm

mh_z19.zero_point_calibration()

while True:

# Detect the sensors that are currently connected

for i in range(0, len(self.sensors)):

try:

self.sensors[i].detect()

self.num_sensors[i] = self.sensors[i].num_sensors

except IOError:

self.logger.info('Error detecting %s sensors',

str(self.sensors[i]))

try:

# Open the sensor readings file and write current timestamp.

self.logger.info('Opening sensors file for records')

self.sensor_readings = codecs.open('sensors.csv','a','utf-8')

self.sensor_readings.write(time.strftime("%Y/%m/%d %H:%M:%S",

time.localtime()))

# Read sensor data from all types of connected sensors.

self.logger.info('Reading sensors from Pi')

total_indoor = 0

total_readings = ""

error_flag = 0

io_flag = 0

for sen in self.sensors:

try:

self.indoor, readings = sen.read()

total_indoor += self.indoor

total_readings += readings

except (IOError, ZeroDivisionError):

self.logger.info('Error reading a sensor.')

error_flag += 1

io_flag = 1

# Read in error and reboot values for updates

self.io_errors = codecs.open('io_error','r','utf-8')

num_errors = int(self.io_errors.read())

self.io_errors.close()

self.reboots = codecs.open('reboots', 'r', 'utf-8')

num_reboots = int(self.reboots.read())

self.reboots.close()

# If maximum reboots not reached, then reboot

if (num_errors >= self.error_max and

num_reboots < self.reboot_max):

self.logger.info('Maximum I/O errors (%d);' +

' rebooting.', num_errors)

self.io_errors = codecs.open('io_error',

'w')

num_reboots += 1

self.io_errors.write('0')

self.io_errors.close()

self.reboots = codecs.open('reboots', 'w')

self.reboots.write((str(num_reboots)))

self.reboots.close()

self.sensor_readings.close()

proc = subprocess.Popen('reboot',

stdout=subprocess.PIPE,

shell=True)

out, err = proc.communicate()

# If maximum reboots reached, stay on

elif num_reboots >= self.reboot_max:

num_errors += 1

self.logger.info('Max reboots (%d) reached;' +

' I/O error #%d occurred',

num_reboots, num_errors)

self.io_errors = codecs.open('io_error',

'w')

self.io_errors.write((str(num_errors)))

self.io_errors.close()

# If maximums not reached, record the error

elif (num_reboots < self.reboot_max and

num_errors < self.error_max):

num_errors += 1

self.logger.info('I/O Error #%d occurred',

num_errors)

self.io_errors = codecs.open('io_error'

'w')

self.io_errors.write((str(num_errors)))

self.io_errors.close()

# No I/O error detected this time -> reset counters

if not io_flag:

self.logger.info('No I/O error detected; ' +

'resetting number of errors '+

'and reboots')

self.io_errors = codecs.open('io_error', 'w')

self.io_errors.write('0')

self.io_errors.close()

self.reboots = codecs.open('reboots', 'w')

self.reboots.write('0')

self.reboots.close()

self.logger.info('Detected indoor temp of %.2f',

total_indoor / len(self.sensors))

# Log the individual readings if we have any sensor data

if error_flag != len(self.sensors):

self.sensor_readings.write(total_readings)

self.logger.info('Reading CO2 data')

try:

# Read CO2 sensor data and log to file

co2_val = mh_z19.read()['co2']

fmt_string = "," + str(co2_val) + "ppm"

self.logger.info('Logging %d ppm to file', co2_val)

self.sensor_readings.write(fmt_string)

except TypeError:

self.logger.info('Unable to read CO2 data')

# Write a new line for the next reading interval

self.sensor_readings.write('\n')

# Close the sensor readings file

self.sensor_readings.close()

# Average temperature readings for accuracy

self.indoor = total_indoor / len(self.sensors)

# Round to three decimal places

self.indoor = round(self.indoor, 3)

self.logger.info('Retrieving outdoor temp from control tent')

# Retrieve outdoor temp from the control tent and parse it

out_proc = subprocess.Popen('scp -o ConnectTimeout=5 pi@' +

self.control_ip +

self.control_dir,

stdout=subprocess.PIPE,

shell=True)

out, err = out_proc.communicate()

try:

out_proc.terminate()

except OSError:

pass

# Open retrieved file, read the line, convert and round.

temp_out = codecs.open('outdoor', 'r')

self.outdoor = float(temp_out.read())

temp_out.close()

self.outdoor = round(self.outdoor, 3)

self.logger.info('Retrieved temperature: %.2f', self.outdoor)

if self.indoor == 0 and self.outdoor == 0:

# both sensors disconnected while running

raise RuntimeError

except RuntimeError as ex:

# Exception occurred with sensor: notify via GUI

self.indoor = 90

self.outdoor = 90

self.heater = "SENSOR"

# Record exception information

self.logger.info('%s', repr(sys.exc_info()))

print((str(ex)))

# If indoor temperature is below differential then

# engage Stage 2 since the purge period and Stage 1

# won't be enough to maintain our differential

if (self.indoor - self.outdoor < self.temperature_diff and

self.indoor != 90 and self.outdoor != 90):

self.heater = "ST2"

GPIO.output(self.signal_pin, GPIO.HIGH)

GPIO.output(self.stage_one_pin, GPIO.HIGH)

GPIO.output(self.stage_two_pin, GPIO.HIGH)

else:

# Indoors >= outdoors -- turn off heater.

if (self.indoor != 90 and self.outdoor != 90):

self.heater = "OFF"

GPIO.output(self.signal_pin, GPIO.LOW)

GPIO.output(self.stage_one_pin, GPIO.LOW)

GPIO.output(self.stage_two_pin, GPIO.LOW)

self.logger.info('%.2f inside, %.2f outside, heater %s',

self.indoor, self.outdoor, self.heater)

# If log interval reached, record the timestamp,

# indoor and outdoor temps, heater status to file

if self.cnt == self.log_interval:

# Log to file every 5 min (60s * 5 = 300s)

self.logger.info('Recording temps data to tent file %s',

self.data_file)

self.output_file = codecs.open(self.data_file, 'a', 'utf-8')

self.output_file.write(repr(self.indoor) +

"," + repr(self.outdoor) + '\n')

self.output_file.close()

self.cnt = 0

# Sleep system until the next check cycle.

time.sleep(self.check_interval)

# Update the counter for the log interval timing

self.logger.info('Incrementing cnt (%d) by check_interval (%d)',

self.cnt, self.check_interval)

self.cnt += self.check_interval

# controlcontroller.py

#!/usr/bin/env python

# Sensor Reading Author: Adafruit Foundation

# Source: https://bit.ly/1iFB8ZP (DS18B20)

# Adapted and modified by: Dan Wagner

# Agronomy Research, 2018-2019

import logging

import sys

import time

import codecs

import subprocess

import mh_z19

class ControlController:

"""

Controller class that manages the Thermostat system

"""

def __init__(self, sensor_list):

"""

Initializes the controller's variables and list of sensors.

"""

# Designate the type of sensor we are using.

self.sensors = sensor_list

# Keep track of the number of each type of sensors connected.

self.num_sensors = [None] * len(self.sensors)

# Filename for specific tent to write data

self.data_file = 'outdoor'

# Format for logging information

self.format = "%(asctime)-15s %(message)s"

# Temperature differential for tent

self.temperature_diff = 4

# Log file interval, in seconds

self.log_interval = 300

# Temperature checking interval, in seconds

self.check_interval = 60

# List of sensors connected to the system

self.sensor_list = []

# Initialize the self.indoor temperature

self.indoor = 0

# Initialize the self.delay time period

self.delay = 0

# Initialize self.heater status to OFF

self.heater = "OFF"

# Initialize counter for time elapsed before logging interval

self.cnt = 0

# Delimit the next day's individual sensor readings via blank line

self.sensor_readings = codecs.open('sensors.csv', 'a', 'utf-8')

self.sensor_readings.write('\n')

self.sensor_readings.close()

# Instantiate the logging for debugging purposes

self.logger = logging.getLogger("Controller")

# Rcord the number of recent I/O errors

self.io_errors = ''

# Record number of reboots the system has experienced

self.reboots = ''

# Maximum number of allowable reboots

self.reboot_max = 5

# Maximum number of allowable I2C errors before reboot

self.error_max = 3

# Main loop of the program.

def main(self):

"""

Configure the logger and record the types of

sensors that have been detected by the controller.

"""

self.logger.basicConfig = logging.basicConfig(format=self.format,

filename='control.log',

level=logging.INFO)

self.logger.info('SYSTEM ONLINE')

# Log the types of sensors we have detected in the system

for sen in self.sensors:

self.logger.info('Detected %s sensors', str(sen))

# Calibrate current CO2 to 410 ppm

mh_z19.zero_point_calibration()

while True:

# Detect the sensors that are currently connected

for i in range(0, len(self.sensors)):

try:

self.sensors[i].detect()

self.num_sensors[i] = self.sensors[i].num_sensors

except IOError:

self.logger.info('Error detecting %s sensors',

str(self.sensors[i]))

try:

# Open the sensor readings file and write current timestamp.

self.logger.info('Opening sensors file for records')

self.sensor_readings = codecs.open('sensors.csv','a','utf-8')

self.sensor_readings.write(time.strftime("%Y/%m/%d %H:%M:%S",

time.localtime()))

# Read sensor data from all types of connected sensors.

self.logger.info('Reading sensors from Pi')

total_indoor = 0

total_readings = ""

error_flag = 0

io_flag = 0

for sen in self.sensors:

try:

self.indoor, readings = sen.read()

total_indoor += self.indoor

total_readings += readings

except (IOError, ZeroDivisionError):

self.logger.info('Error reading a sensor.')

error_flag += 1

io_flag = 1

# Read in error and reboot values for updates

self.io_errors = codecs.open('io_error','r','utf-8')

num_errors = int(self.io_errors.read())

self.io_errors.close()

self.reboots = codecs.open('reboots','r','utf-8')

num_reboots = int(self.reboots.read())

self.reboots.close()

# If maximum reboots not reached, then reboot

if (num_errors >= self.error_max and

num_reboots < self.reboot_max):

self.logger.info('Maximum I/O errors (%d);' +

' rebooting.', num_errors)

self.io_errors = codecs.open('io_error','w')

num_reboots += 1

self.io_errors.write('0')

self.io_errors.close()

self.reboots = codecs.open('reboots', 'w')

self.reboots.write((str(num_reboots)))

self.reboots.close()

self.sensor_readings.close()

proc = subprocess.Popen('reboot',

stdout=subprocess.PIPE,

shell=True)

out, err = proc.communicate()

# If maximum reboots reached, stay on

elif num_reboots == self.reboot_max:

num_errors += 1

self.logger.info('Max reboots (%d) reached;' +

' I/O error #%d occurred',

num_reboots, num_errors)

self.io_errors = codecs.open('io_error',

'w')

self.io_errors.write((str(num_errors)))

self.io_errors.close()

# If maximums not reached, record the error

elif (num_reboots < self.reboot_max and

num_errors < self.error_max):

num_errors += 1

self.logger.info('I/O Error #%d occurred',

num_errors)

self.io_errors = codecs.open('io_error'

'w')

self.io_errors.write((str(num_errors)))

self.io_errors.close()

# No I/O error detected this time -> reset counters

if not io_flag:

self.logger.info('No I/O error detected; ' +

'resetting number of errors '

+ 'and reboots')

self.io_errors = codecs.open('io_error', 'w')

self.io_errors.write('0')

self.io_errors.close()

self.reboots = codecs.open('reboots', 'w')

self.reboots.write('0')

self.reboots.close()

self.logger.info('Detected indoor temp of %.2f',

total_indoor / len(self.sensors))

# Log the individual readings if we have any sensor data

if error_flag != len(self.sensors):

self.sensor_readings.write(total_readings)

self.logger.info('Reading CO2 data')

try:

# Read CO2 sensor data and log to file

co2_val = mh_z19.read()['co2']

fmt_string = "," + str(co2_val) + "ppm"

self.logger.info('Logging %d ppm to file', co2_val)

self.sensor_readings.write(fmt_string)

except TypeError:

self.logger.info('Unable to read CO2 data')

# Write a new line for the next reading interval

self.sensor_readings.write('\n')

# Close the sensor readings file

self.sensor_readings.close()

# Average temperature readings for accuracy

self.indoor = total_indoor / len(self.sensors)

# Round to three decimal places

self.indoor = round(self.indoor, 3)

if self.indoor == 0:

# sensors disconnected while running

raise RuntimeError

except RuntimeError as ex:

# Exception occurred with sensors

self.indoor = 90

self.heater = "SENSOR"

# Record exception information

self.logger.info('%s', repr(sys.exc_info()))

print((str(ex)))

# Immediately record outdoor temperature to file for control

self.logger.info('Control: %d outside', self.indoor)

self.logger.info('Recording temperature data to tent file %s',

self.data_file)

if self.indoor != 90:

self.output_file = codecs.open(self.data_file, 'w', 'utf-8')

self.output_file.write(repr(self.indoor))

self.output_file.close()

else:

self.logger.info('Cannot read sensors. No temperature data.')

time.sleep(self.check_interval)
