# Supplementary figures and images for: Improved cyber-physical system captured post-flowering high night temperature impact on yield and quality of field grown wheat
Source: Sci Rep. 2020 Dec 17;10:22213. doi: 10.1038/s41598-020-79179-0 (PMC7747627; doi:10.1038/s41598-020-79179-0)

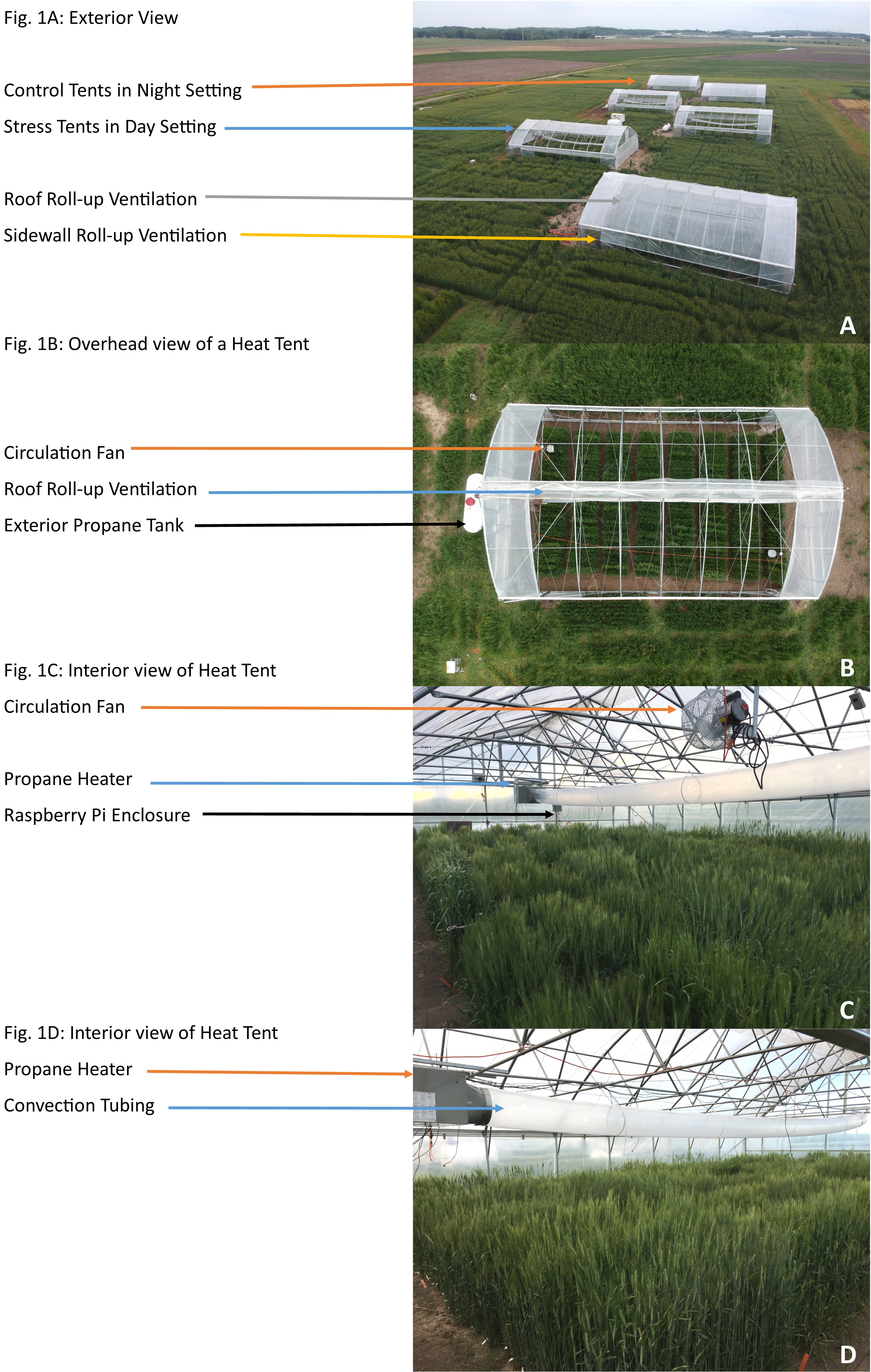

Supplement: Supplementary file 3 — Supplementary Figure 1. [file 41598_2020_79179_MOESM3_ESM.jpg]

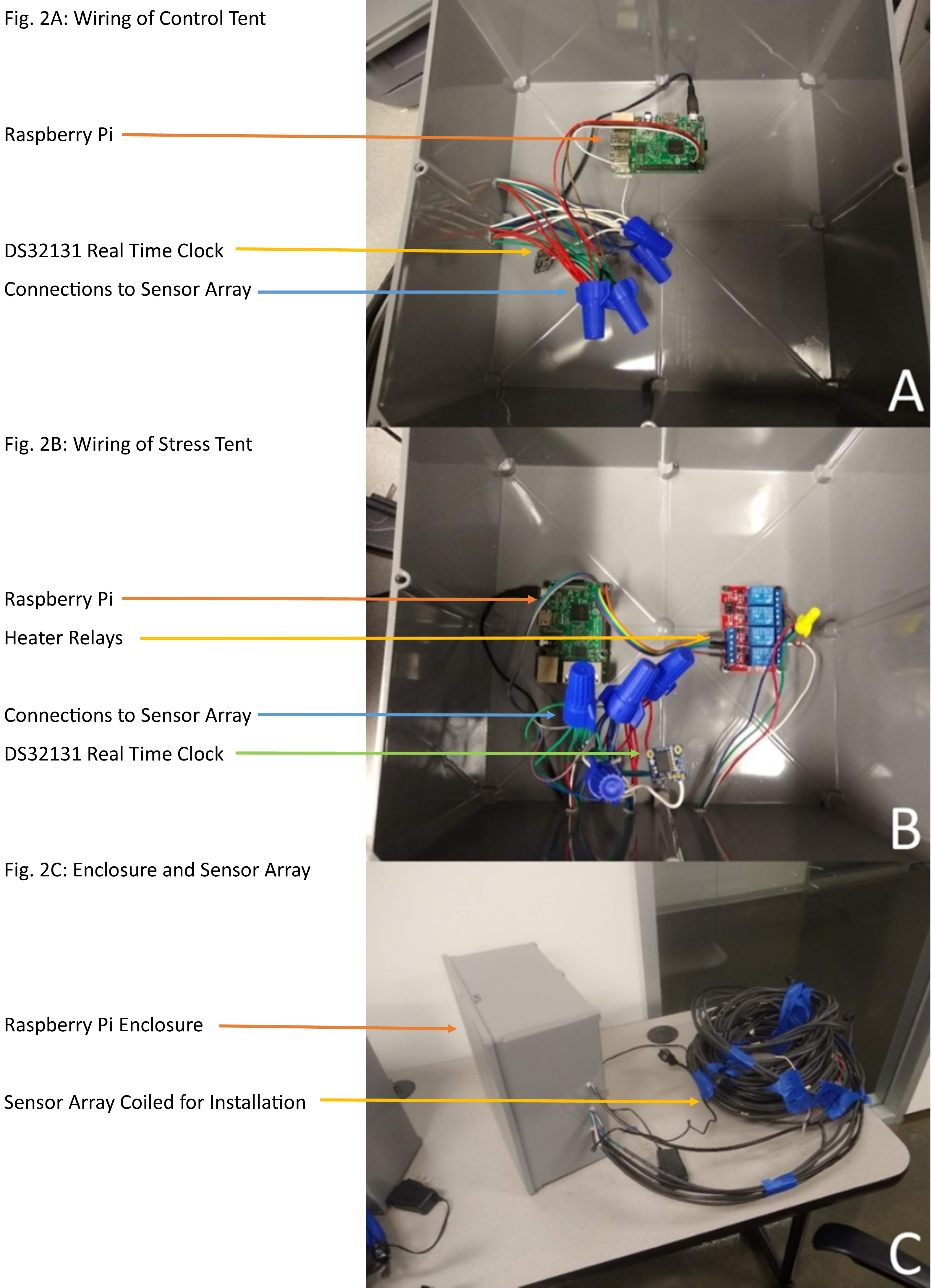

Supplement: Supplementary file 4 — Supplementary Figure 2. [file 41598_2020_79179_MOESM4_ESM.jpg]

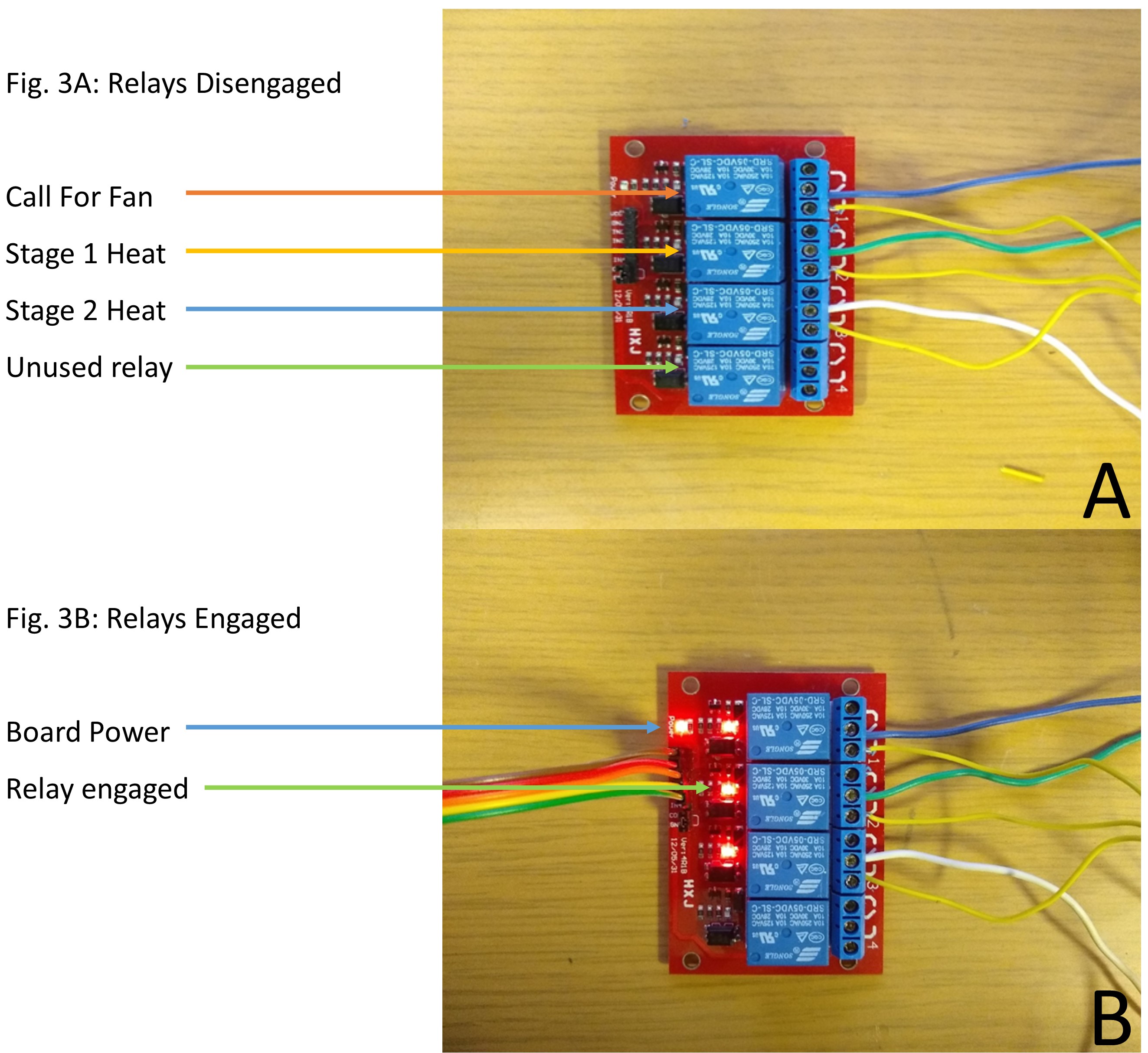

Supplement: Supplementary file 5 — Supplementary Figure 3. [file 41598_2020_79179_MOESM5_ESM.jpg]

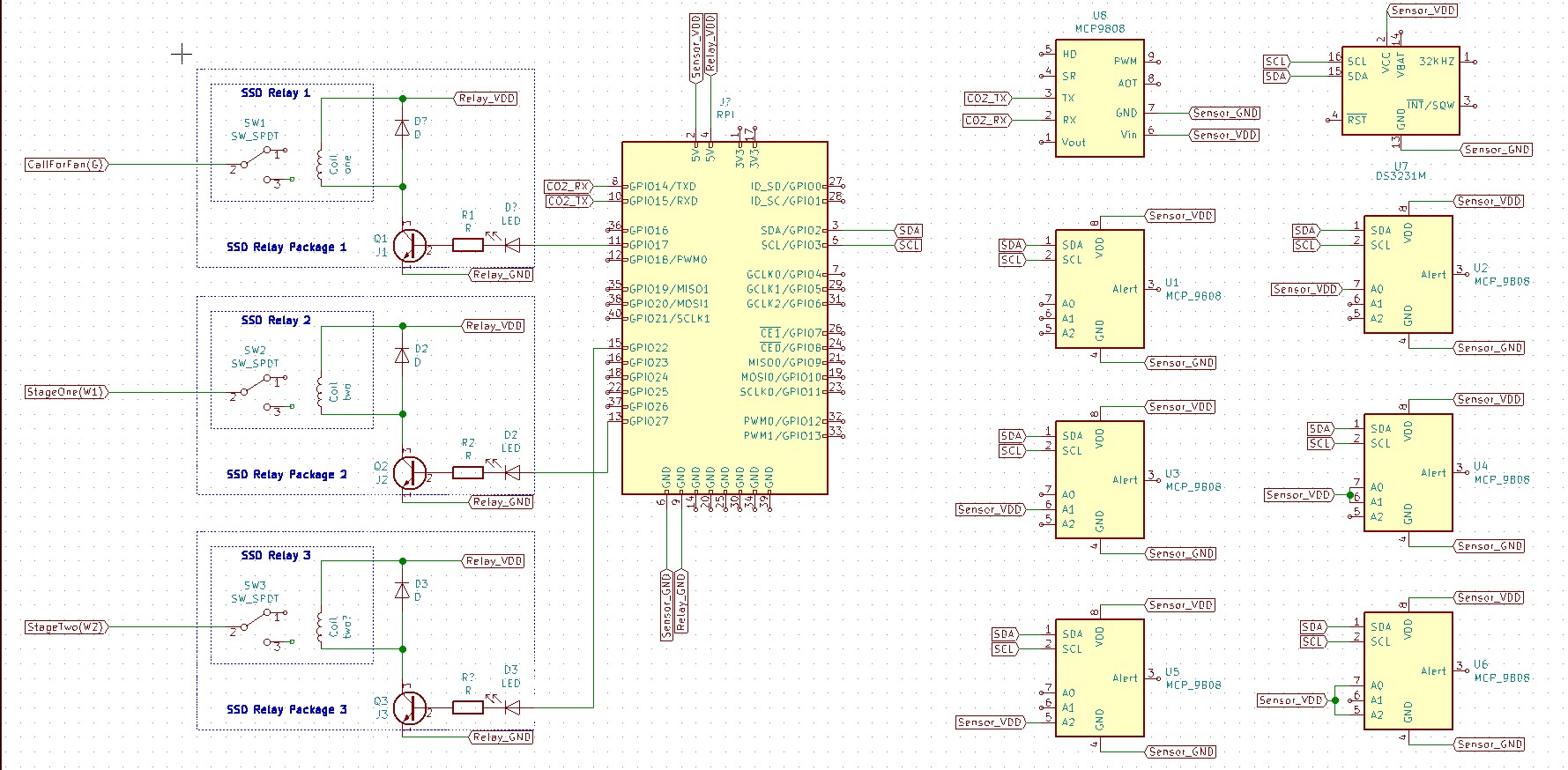

Supplement: Supplementary file 6 — Supplementary Figure 4. [file 41598_2020_79179_MOESM6_ESM.jpg]

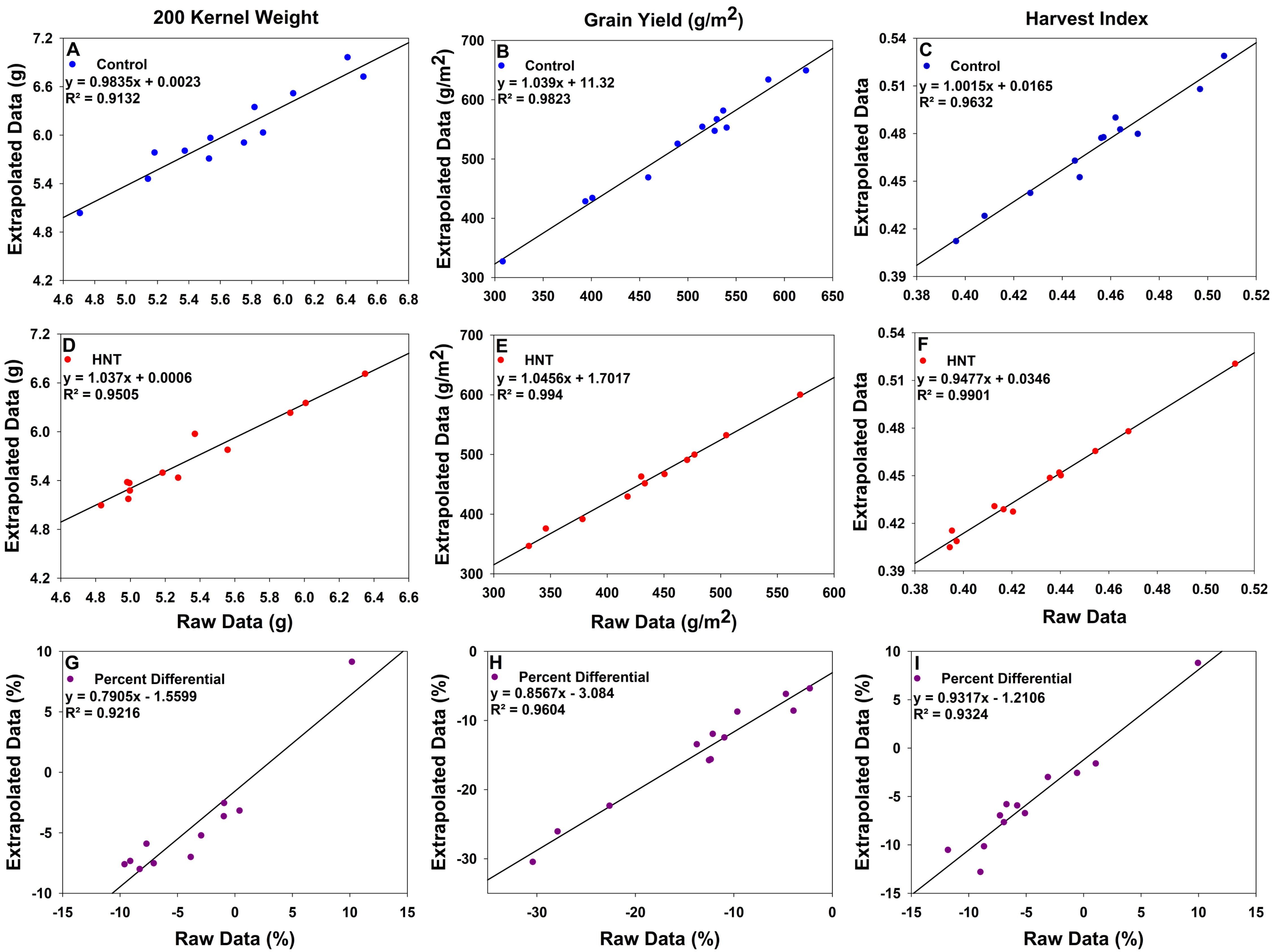

Supplement: Supplementary file 7 — Supplementary Figure 5. [file 41598_2020_79179_MOESM7_ESM.jpg]

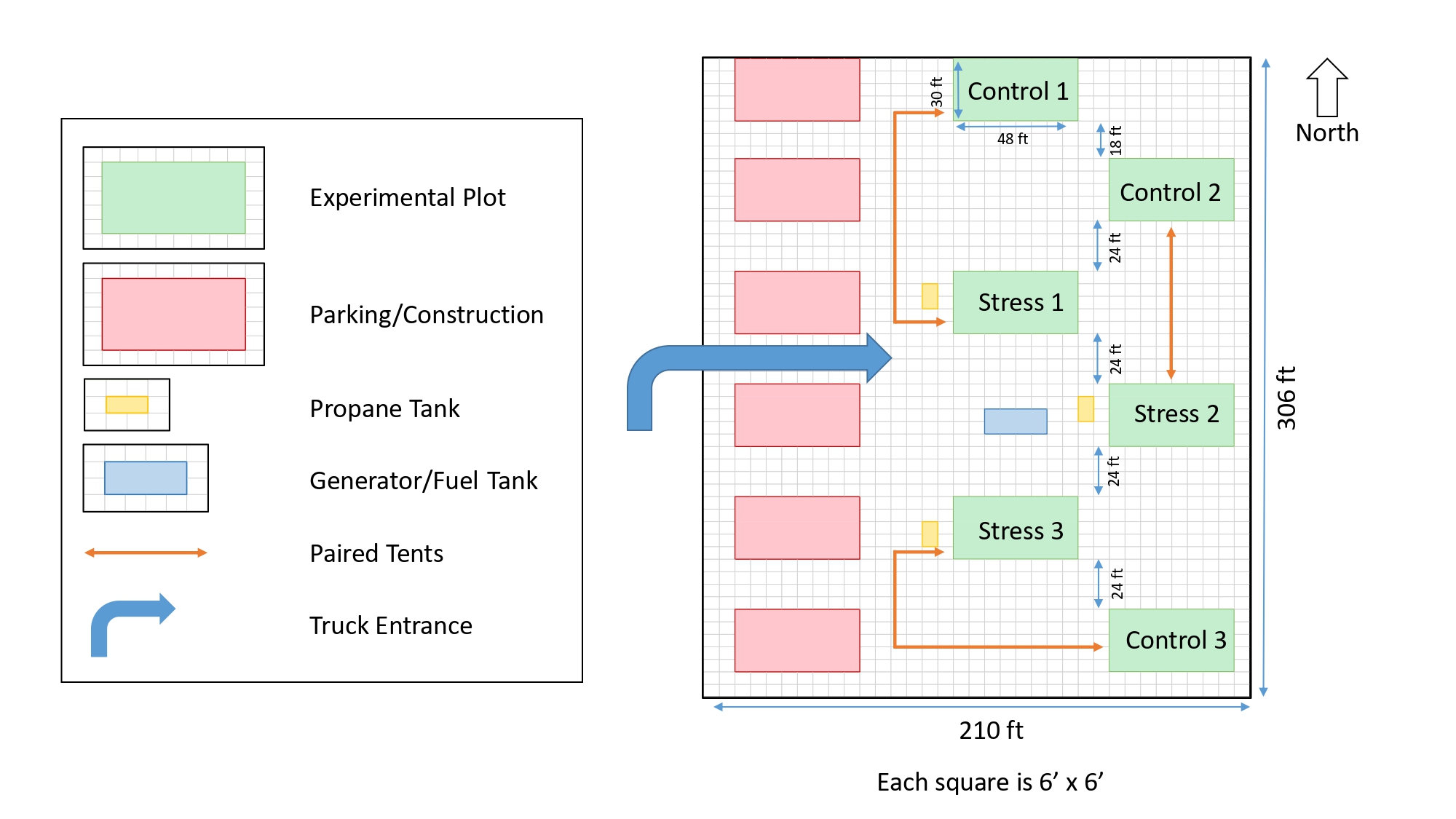

Supplement: Supplementary file 8 — Supplementary Figure 6. [file 41598_2020_79179_MOESM8_ESM.jpg]

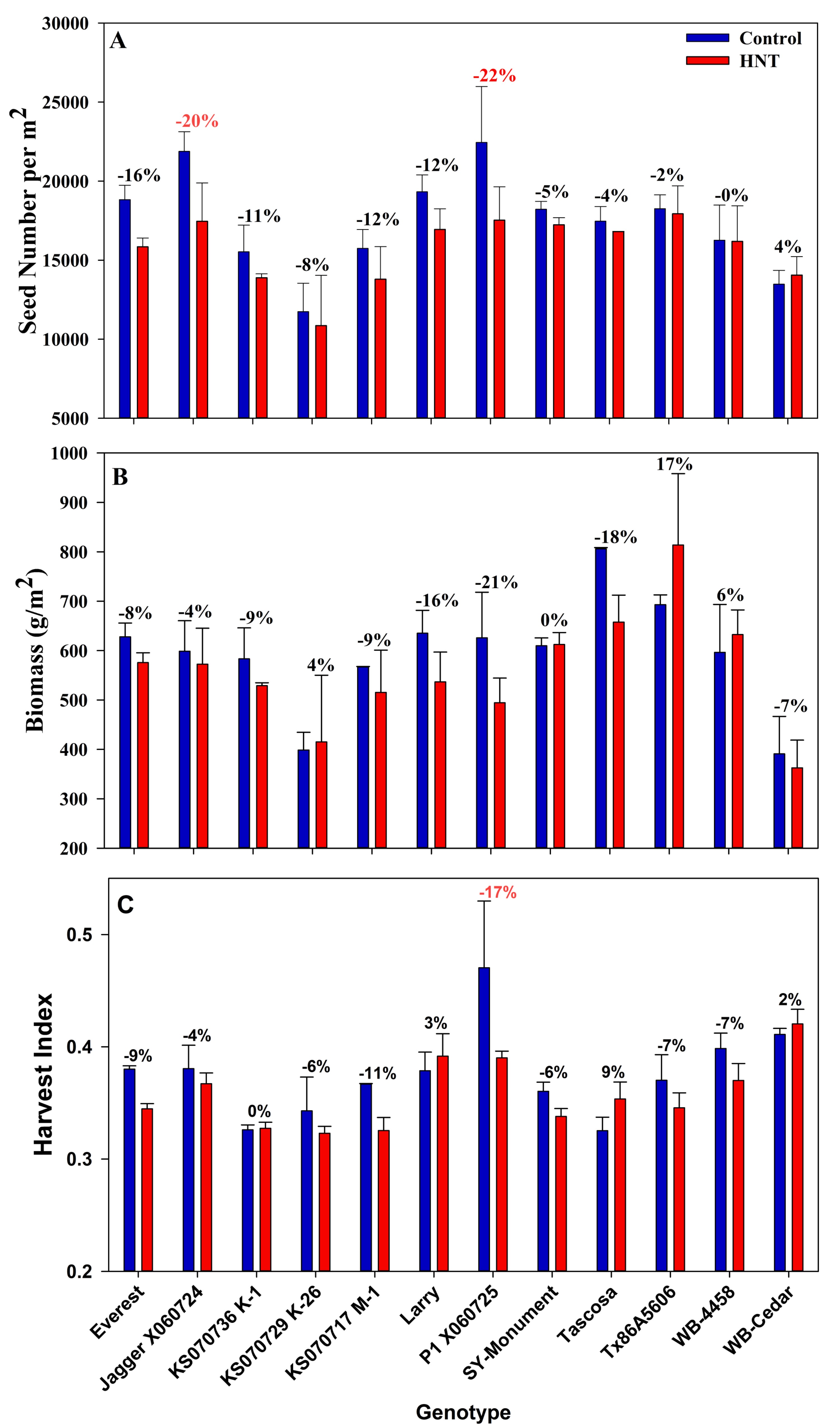

Supplement: Supplementary file 9 — Supplementary Figure 7. [file 41598_2020_79179_MOESM9_ESM.jpg]
